# Supplementary material for: Integration of clinical, pathological, radiological, and transcriptomic data improves prediction for first-line immunotherapy outcome in metastatic non-small cell lung cancer
Source: Nat Commun. 2025 Jan 12;16:614. doi: 10.1038/s41467-025-55847-5 (PMC11725576; doi:10.1038/s41467-025-55847-5)
Supplement: Supplementary file 1 — Supplementary Information [file 41467_2025_55847_MOESM1_ESM.pdf]

Supplementary Information for:

"Integration of clinical, pathological, radiological, and transcriptomic data improves prediction for first-line immunotherapy outcome in metastatic non-small cell lung cancer"

Authors and affiliations:

Nicolas Captier[1,2*], Marvin Lerousseau[2,3], Fanny Orlhac[1], Narinée Hovhannisyan-Baghdasarian[1], Marie Luporsi[1,4], Erwin Woff[1,5], Sarah Lagha[6], Paulette Salamoun Feghali[6], Christine Lonjou[2], Clément Beaulaton[7], Andrei Zinovyev[8], Hélène Salmon[9], Thomas Walter[2,3#], Irène Buvat[1#], Nicolas Girard[6#], Emmanuel Barillot[2*#]

1: Laboratoire d'Imagerie Translationnelle en Oncologie, Institut Curie, Inserm U1288, PSL Research University, Orsay, France

2: Bioinformatics and computational systems biology of cancer, Institut Curie, Inserm U900, PSL Research University, Paris, France

3: CBIO-center for Computational Biology, MINES ParisTech, PSL Research University, Paris, France

4: Department of medical imaging, Institut Curie, Paris, France

5: Department of Nuclear Medicine/PET-scan, Institut Jules Bordet, Université Libre de Bruxelles, Brussels, Belgium

6: Institut du Thorax Curie-Montsouris, Institut Curie, Paris, France

7: Department of pathology, Institut Curie, Paris, France

8: In silico R&D, Evotec, Toulouse, France

9: Immunity and cancer, Institut Curie, Inserm U932, PSL Research University, Paris, France

*** - corresponding authors:**

Nicolas Captier - nicolas.captier@polytechnique.org

Emmanuel Barillot - emmanuel.barillot@curie.fr

# - Joint supervision

The supplementary information file is organized as follows:

- Page 3 to 5: Supplementary Methods with glossaries for clinical and radiomic features
- Page 6 to 36: Supplementary Figures 1 to 24
- Page 37 to 38: Supplementary Tables 1 and 2
- Page 39: Supplementary References

Supplementary Methods

Glossary of clinical features

- **age:** Age of the patient at the diagnosis of the metastatic disease.
- **albumin:** Serum albumin (g/l).
- **alk:** Detected ALK mutation prior to the initiation of first-line therapy (1: detected, 0: otherwise).
- **asat:** Serum Aspartate Aminotransferase (AST) (IU/l).
- **bmi:** Body Mass Index (kg/m^2) at the diagnosis of the metastatic disease.
- **braf:** Detected BRAF mutation prior to the initiation of first-line therapy (1: detected, 0: otherwise).
- **chemotherapy:** Whether the patient received chemotherapy+pembrolizumab or not (1: pembrolizumab+chemotherapy, 0: pembrolizumab alone).
- **ecog:** ECOG status (Eastern Cooperative Oncology Group) at the diagnosis of the metastatic disease, one-hot encoded into 4 binary features (ecog_0, ecog_1, ecog_2, ecog_4).
- **egfr:** Detected EGFR mutation prior to the initiation of first-line therapy (1: detected, 0: otherwise).
- **erbb2:** Detected ERBB2 mutation prior to the initiation of first-line therapy (1: detected, 0: otherwise).
- **height:** Height of the patient (cm).
- **histo:** Histology one-hot encoded into 4 binary features (histo_adeno for adenocarcinomas, histo_squamous for squamous cell carcinomas, histo_other-nsclc for other NSCLC subtypes, and histo_other for other unspecified subtypes).
- **kras:** Detected KRAS mutation prior to the initiation of first-line therapy (1: detected, 0: otherwise).
- **ldh:** Serum Lactate dehydrogenase (LDH) (IU/l).
- **lung_surgery:** Whether the patient had a lung surgery prior to the first-line therapy (1: prior lung surgery, 0: otherwise).
- **lymphocytes:** Circulating lymphocytes count (10e9/l).
- **meta_brain:** Whether a brain metastasis was detected prior to the initiation of first-line therapy (1: detected brain metastasis, 0: otherwise).
- **met:** Detected MET mutation prior to the initiation of first-line therapy (1: detected, 0: otherwise).
- **neutrophils:** Circulating neutrophils count (10e9/l).
- **neutrophils/lymphocytes:** Neutrophils-to-lymphocytes ration (NLR).
- **n_preceding_cancers**: Number of preceding cancers.
- **other_mutations:** Detected mutations (different from BRAF, EGFR, ERBB2, KRAS, MET, or ROS1) prior to the initiation of first-line therapy (1: detected, 0: otherwise)
- **pack_years:** Smoking history in pack-years (i.e., equivalent of smoking one pack of 20 cigarettes a day for one year).
- **pdl1:** PD-L1 status (1: positive detection of PD-L1 expression with immunohistochemistry prior to the initiation of first-line therapy, 0: otherwise). If several tests were performed before the first-line therapy a positive test prevails over negative tests.
- **pdl1_tps:** PD-L1 Tumor Proportion Score (TPS). If several tests were performed before the initiation of first-line therapy, the maximum score across the tests is chosen. For patient with negative PD-L1 status this feature is set to 0.

- **ros1:** Detected ROS1 mutation prior to the initiation of first-line therapy (1: detected, 0: otherwise).
- **sex:** Sex of the patient (0: woman, 1: man).
- **smoking:** Smoking status with 3 categories (i.e., 3 binary variables), smoking_never, smoking_past, and smoking_current.
- **tils:** Detection of Tumor-Infiltrating Lymphocytes (TILs) prior to the initiation of first-line therapy (1: positive detection, 0: otherwise).
- **weight:** Weight of the patient at the diagnosis of the metastatic disease (kg).

Glossary of radiomics features

- **Distance dispersion:** Quartile dispersion of the distances between each tumor region's centroid and the global centroid.
- **Dmax:** Largest distance between the centroids of two lesions normalized by the body surface area.
- **liver_SUVMean:** Mean of SUV values in a spherical ROI manually delineated in a healthy part of the liver on the PET scan.
- **Nb invaded organs:** Number of invaded organs visible on the PET scan, including the lungs, sub- and supra-diaphragmatic lymph nodes, the pleura, the liver, the bones, the adrenal gland, and a final category for other regions.
- **N1_Suvmax_max/mean/std:** Maximum/Mean/Standard deviation value of the SUVmax of the segmented lesions located in regions associated with the N1 stage (i.e., ipsilateral hilar and mediastinal-hilar lymph nodes). 0 if no lesions in these regions.
- **N1_TMTV:** Total Metabolic Tumor Volume computed with the segmented lesions located in regions associated with the N1 stage. 0 if no lesions in these regions.
- **N2_Suvmax_max/mean/std:** Maximum/Mean/Standard deviation value of the SUVmax of the segmented lesions located in regions associated with the N2 stage (i.e., ipsilateral mediastinal lymph nodes and subcarinal lymph nodes). 0 if no lesions in these regions.
- **N2_TMTV:** Total Metabolic Tumor Volume computed with the segmented lesions located in regions associated with the N2 stage. 0 if no lesions in these regions.
- **N3_Suvmax_max/mean/std:** Maximum/Mean/Standard deviation value of the SUVmax of the segmented lesions located in regions associated with the N3 stage (i.e., contralateral mediastinal and hilar lymph nodes and supraclavicular lymph nodes). 0 if no lesions in these regions.
- **N3_TMTV:** Total Metabolic Tumor Volume computed with the segmented lesions located in regions associated with the N3 stage. 0 if no lesions in these regions.
- **M1a_Suvmax_max/mean/std:** Maximum/Mean/Standard deviation value of the SUVmax of the segmented lesions located in regions associated with the M1a stage (i.e., contralateral lung metastases and pleural metastases). 0 if no lesions in these regions.
- **M1a_TMTV:** Total Metabolic Tumor Volume computed with the segmented lesions located in regions associated with the M1a stage (excluding diffuse pleural metastases). 0 if no lesions in these regions.
- **M1bc_Suvmax_max/mean/std:** Maximum/Mean/Standard deviation value of the SUVmax of the segmented lesions located in regions associated with the M1b and M1c stages (i.e., extrathoracic metastases). 0 if no lesions in these regions.
- **M1bc_TMTV:** Total Metabolic Tumor Volume computed with the segmented lesions located in regions associated with the M1b and M1c stages (excluding diffuse subdiaphragmatic metastases). 0 if no lesions in these regions.

- **spleen_SUVMean:** Mean of SUV values in a spherical ROI manually delineated in a healthy part of the spleen on the PET scan.
- **TMTV:** Total Metabolic Tumor Volume computed with all the segmented lesions (excluding diffuse lesions corresponding to lymphangitic spread, diffuse pleural metastases, diffuse myocardial metastases, and diffuse subdiaphragmatic metastases).
- **T_Suvmax_max/mean/std:** Maximum/Mean/Standard deviation value of the SUVmax of the segmented lesions located in regions associated with the T stage (i.e., primary lung tumor and ipsilateral lung metastases). 0 if no lesions in these regions.
- **T_TMTV:** Total Metabolic Tumor Volume computed with the segmented lesions located in regions associated with the T stage (excluding lymphangitic spread). 0 if no lesions in these regions.

Supplementary Figures

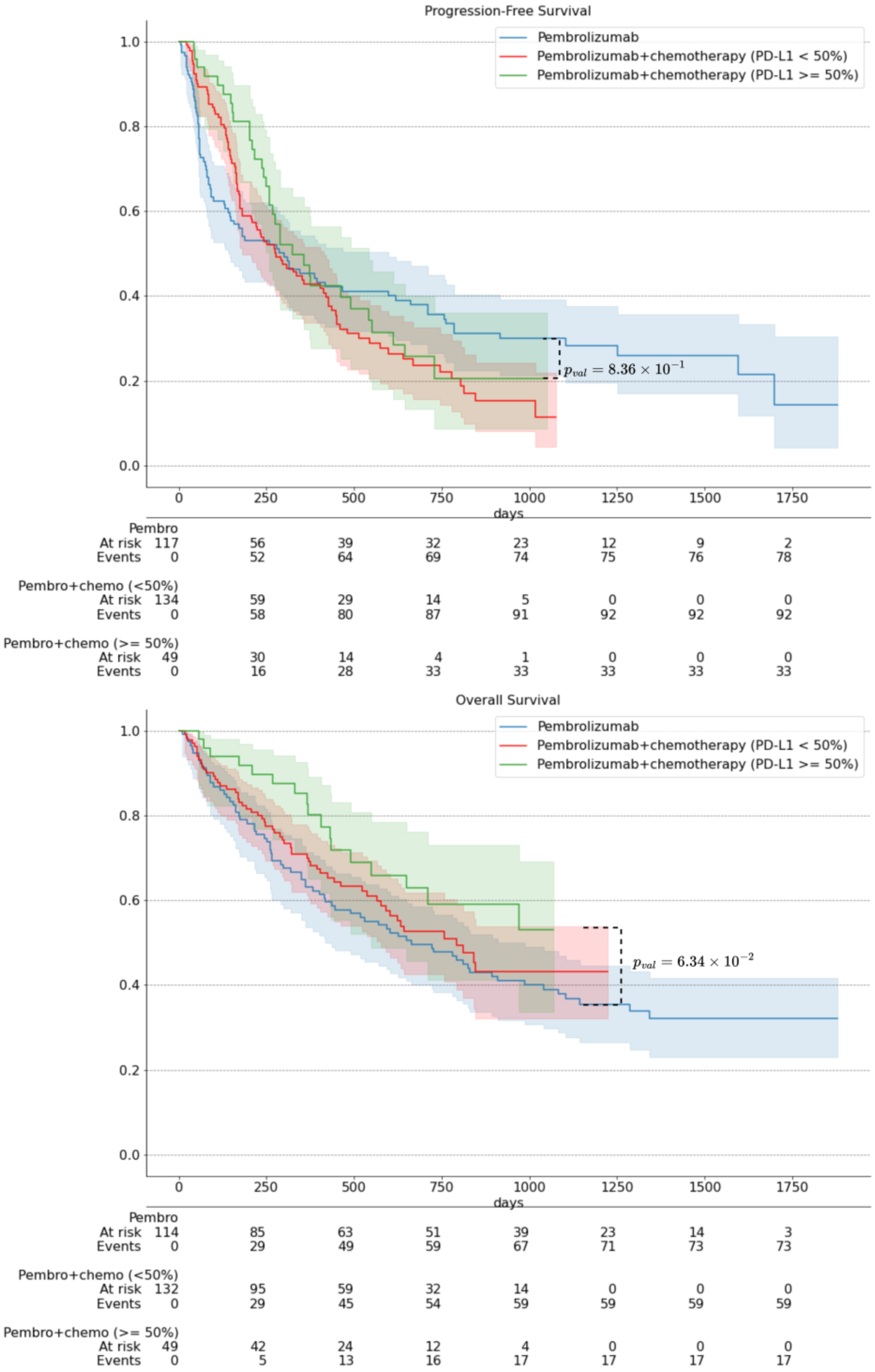

Figure s1: Survival of NSCLC patients stratified by treatment group and PD-L1 expression.

OS and PFS Kaplan-Meier survival curves (solid lines) for patients with available PD-L1 expression (n=295 for OS and n=300 for PFS) with 95% confidence interval (shaded areas). Patients are first stratified with respect to their first-line therapy (pembrolizumab vs pembrolizumab + chemotherapy). Patients treated with pembrolizumab + chemotherapy are also stratified with respect to their PD-L1 expression (PD-L1 TPS < 50% vs PD-L1 TPS ≥ 50%). Log-rank p-values are reported to assess the separation of survival curves.

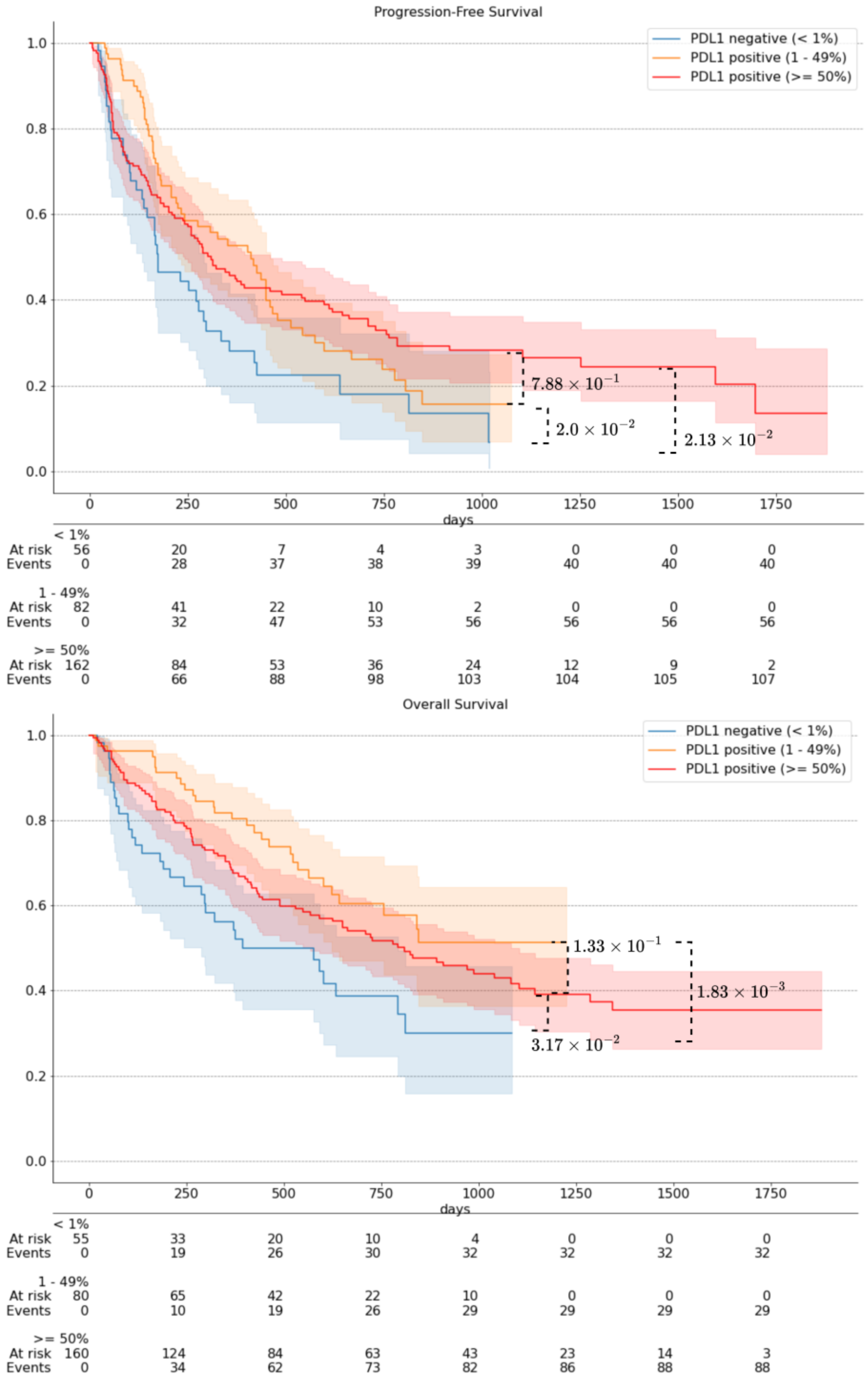

Figure s2: Survival of NSCLC patients stratified by PD-L1 expression.

OS and PFS Kaplan-Meier survival curves (solid lines) for patients with available PD-L1 expression (n=295 for OS and n=300 for PFS) with 95% confidence interval (shade areas). Patients are stratified with respect to PD-L1 expression (PD-L1 negative (i.e., TPS < 1%) vs PD-L1 positive with TPS < 50% vs PD-L1 positive with TPS ≥ 50%). Log-rank p-values are reported to assess the separation of the survival curves.

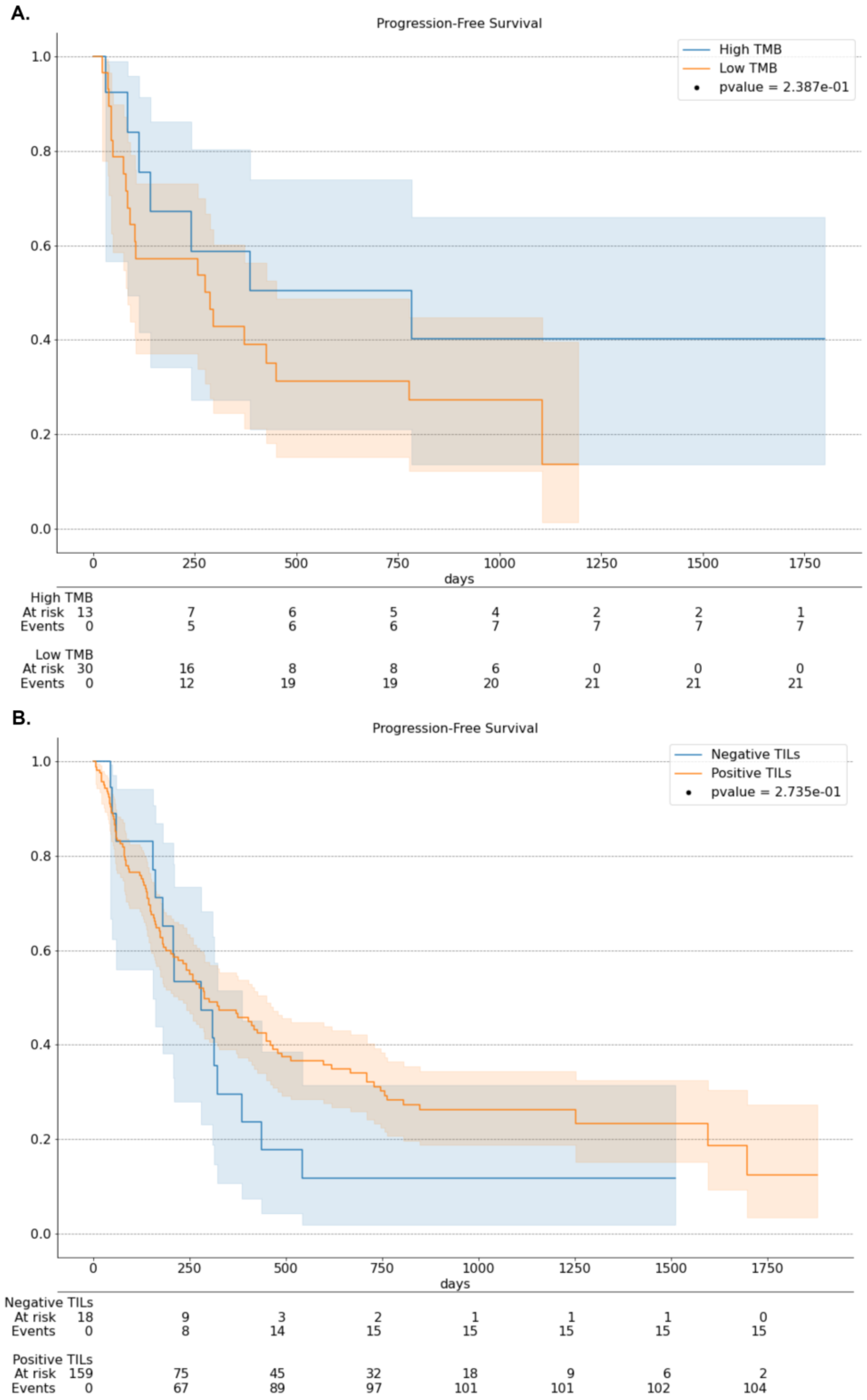

Figure s3: Survival of NSCLC patients stratified by TMB value and TILs status.

A. PFS Kaplan-Meier survival curves (solid lines) with 95% confidence interval (shaded areas) for the 43 patients with available TMB. Patients are stratified with a threshold of 15 mutations per megabase. Log-rank p-values as well as the number of patients at risk are reported to assess the separation of the survival curves. **B.** PFS Kaplan-Meier survival curves with 95% confidence interval for the 177 patients with available TILs status. Patients are stratified with respect to their positive vs negative TILs status. Log-rank p-values as well as the number of patients at risk are reported to assess the separation of the survival curves.

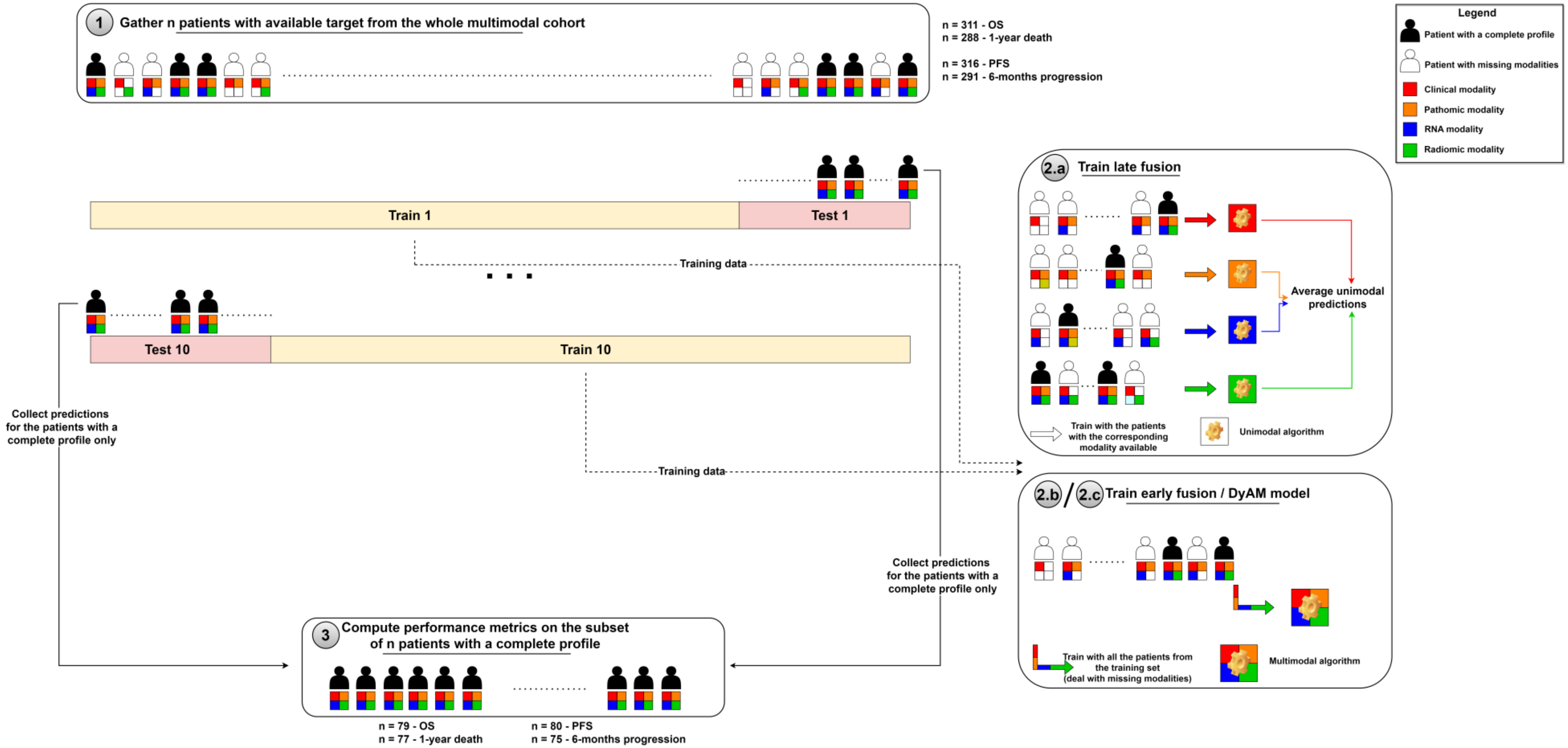

Figure s4: Train and test pipeline for all the predictive models.

Here the combination with 4 modalities is represented but this scheme is valid for every combination (removing patients from the training set with no modalities available). **1.** All the patients with available target (i.e., OS, 1-year death, PFS, or 6-months progression) are collected from the whole multimodal cohort (n=317), they are shuffled and a stratified 10-fold cross-validation scheme is applied. **2.** For each fold, data from the training set are used to train the predictive model (pre-processing + learning algorithm), whether it is a unimodal model, a late fusion model, an early fusion model or a DyAM model. The trained predictive model is then applied to the test set of the corresponding fold. **3.** Predictions from each test set are collected, considering only those made for the patients with a complete profile. Lastly, performance metrics are computed with this subset of collected predictions.

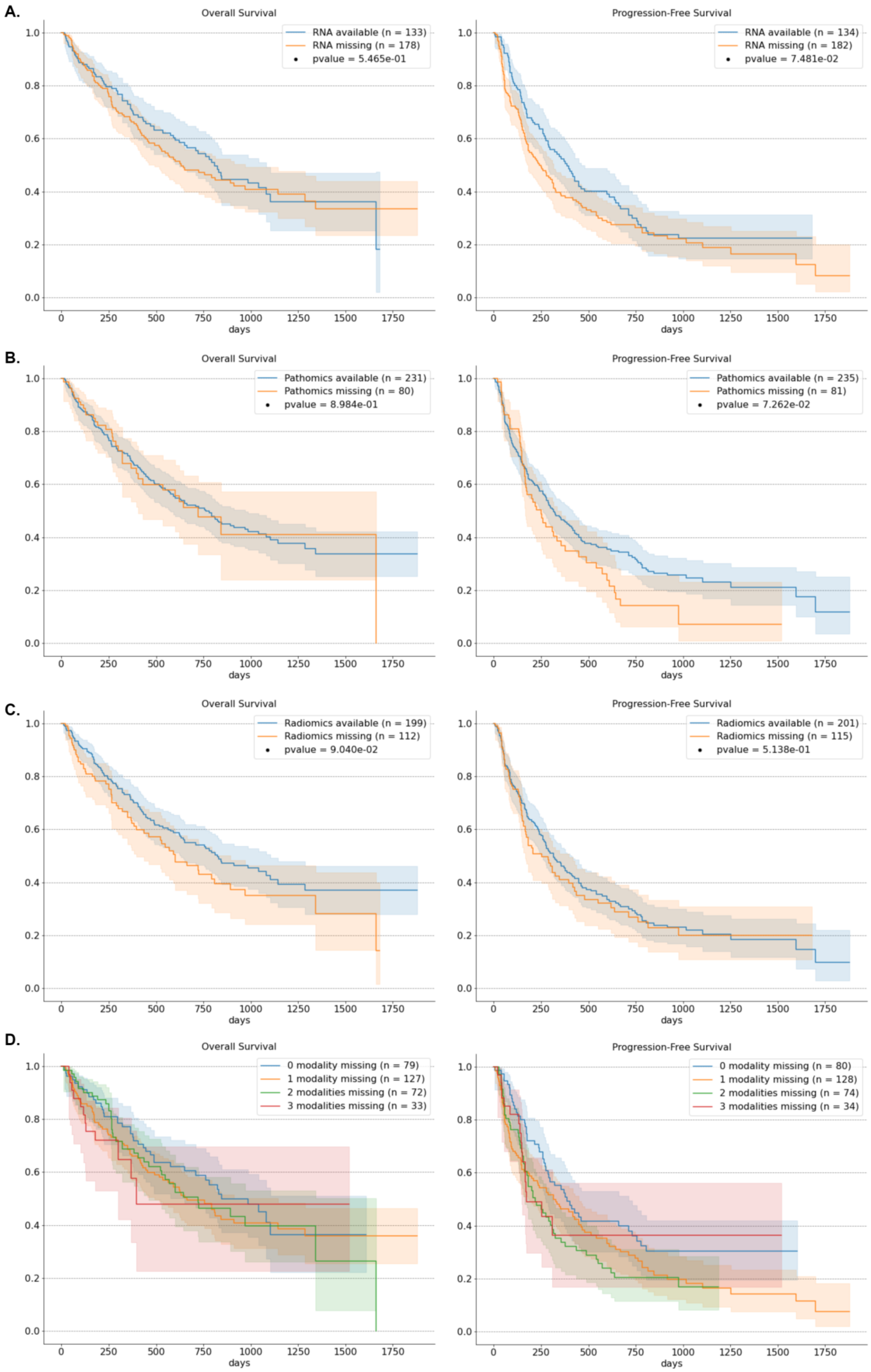
A.
Overall Survival
RNA available (n = 133)
RNA missing (n = 178)
pvalue = 5.465e-01
Progression-Free Survival
RNA available (n = 134)
RNA missing (n = 182)
pvalue = 7.481e-02
B.
Overall Survival
Pathomics available (n = 231)
Pathomics missing (n = 80)
pvalue = 8.984e-01
Progression-Free Survival
Pathomics available (n = 235)
Pathomics missing (n = 81)
pvalue = 7.262e-02
C.
Overall Survival
Radiomics available (n = 199)
Radiomics missing (n = 112)
pvalue = 9.040e-02
Progression-Free Survival
Radiomics available (n = 201)
Radiomics missing (n = 115)
pvalue = 5.138e-01
D.
Overall Survival
0 modality missing (n = 79)
1 modality missing (n = 127)
2 modalities missing (n = 72)
3 modalities missing (n = 33)
Progression-Free Survival
0 modality missing (n = 80)
1 modality missing (n = 128)
2 modalities missing (n = 74)
3 modalities missing (n = 34)
days

Figure s5: Survival of NSCLC patients with missing modalities.

OS and PFS Kaplan-Meier survival curves (solid lines) with 95% confidence interval (shaded areas) for the whole NSCLC cohort (n=311 for OS and n=316 for PFS). Log-rank p-values are reported to assess the separation of survival curves. **A.** Patients are stratified with respect to the availability of RNA data. **B.** Patients are stratified with respect to the availability of pathomics data. **C.** Patients are stratified with respect to the availability of radiomics data. **D.** Patients are stratified with respect to the number of missing modalities.

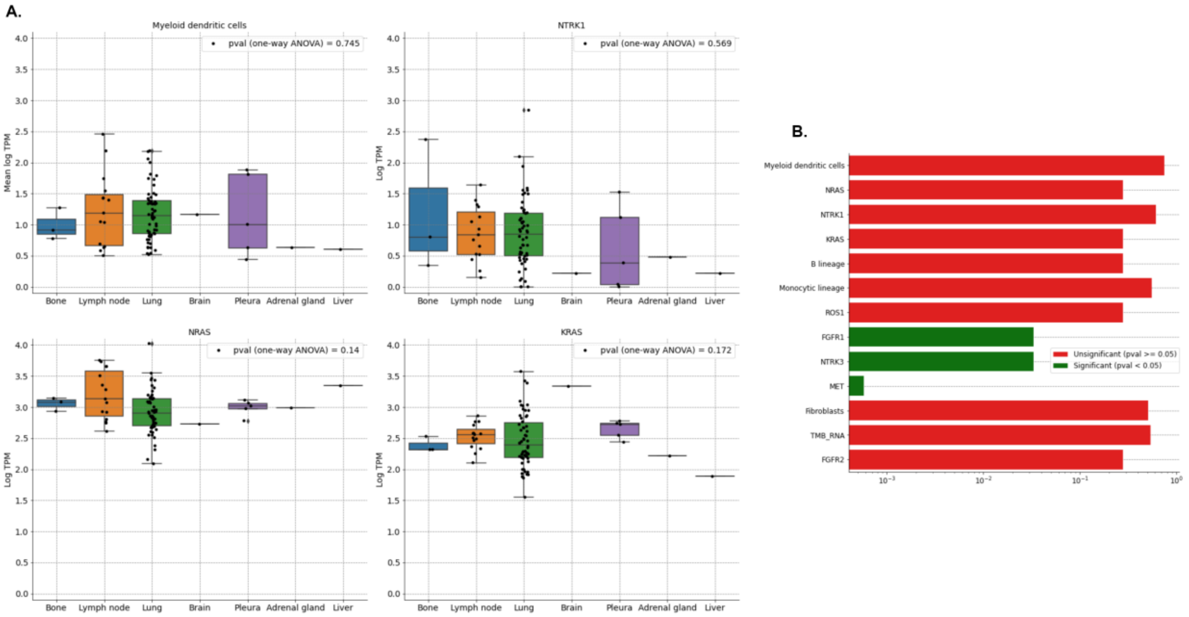

Figure s6: Value of consensus transcriptomic features for different biopsy sites.

Association between the consensus transcriptomic features (i.e., involved in the prediction of overall survival) and the biopsy site (n=84 patients). One-way ANOVA tests are used to test for statistically significant differences between the means of the transcriptomic feature in the different biopsy sites. **A.** Distribution of the four most important transcriptomic features in the different biopsy sites. The box-and-whisker plots show the three quartiles and the minimum and maximum as whiskers up to $1.5 \times IQR$ (25%-75%). **B.** One-way ANOVA p-values for each of the 13 consensus transcriptomic features, displayed with a bar plot in log-scale. P-values were corrected with Benjamini-Hochberg procedure (FDR controlled at level $\alpha = 0.05$).

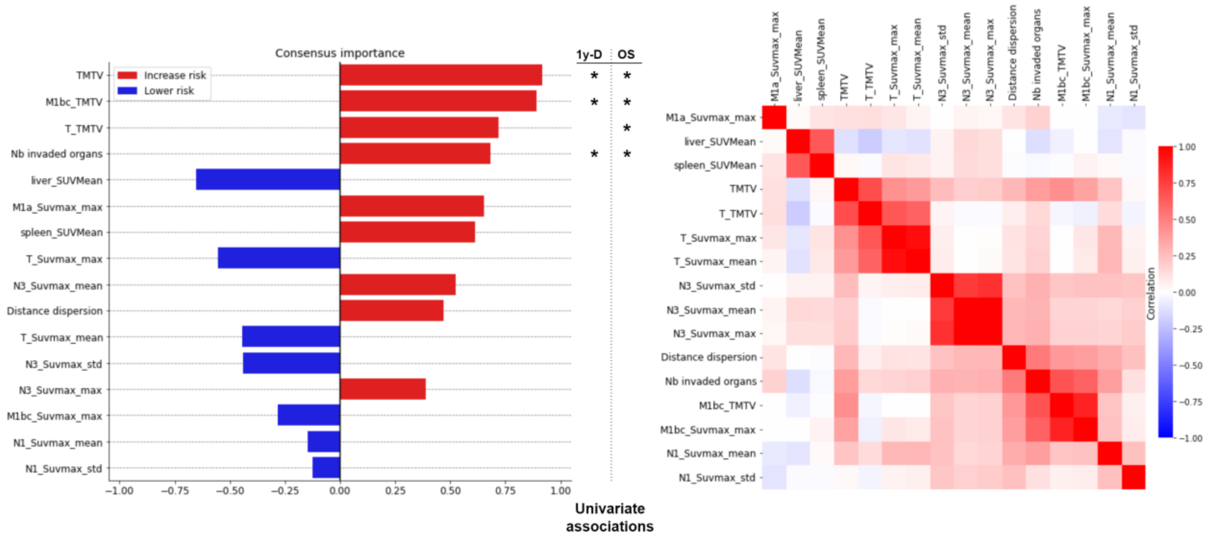

Figure s7: Feature importance ranking for the prediction of overall survival, for radiomic modality.

Feature importance ranking for the prediction of overall survival (OS and 1-year death) with radiomic modality (left) and heatmap of Spearman correlations between consensus radiomic features (right). Logistic regression was not taken into account for computing this ranking since its AUC was lower than 0.5. Features that were significantly associated with 1-year death (one-sided permutation test with univariate AUCs) after Benjamini-Hochberg (BH) correction ($\alpha = 0.05$) are annotated with a * on the left side, while features that were significantly associated with OS (permutation test with univariate C-index) after BH correction are annotated with a * on the right side. * corresponds to an adjusted p-value below 0.05.

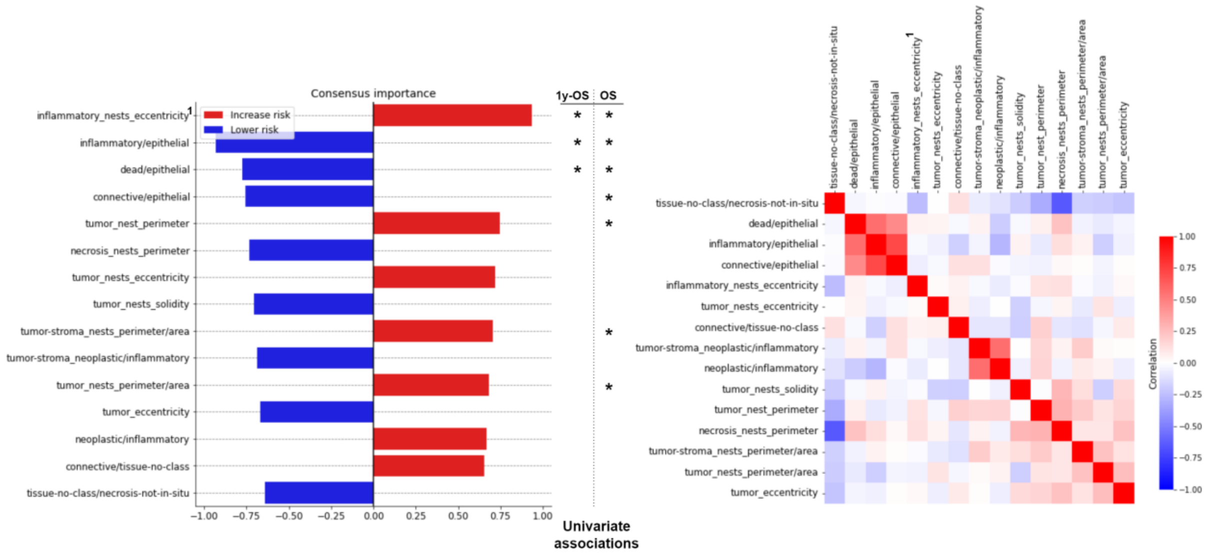

1. **inflammatory_nests_eccentricity:** eccentricity of the largest inflammatory nest

Figure s8: Feature importance ranking for the prediction of overall survival, for pathomic modality.

Feature importance ranking for the prediction of overall survival (OS and 1-year death) with pathomic modality (left) and heatmap of Spearman correlations between consensus pathomic features (right). Features that were significantly associated with 1-year death (one-sided permutation test with univariate AUCs) after Benjamini-Hochberg (BH) correction ($\alpha = 0.05$) are annotated with a * on the left side, while features that were significantly associated with OS (permutation test with univariate C-index) after BH correction are annotated with a * on the right side. * corresponds to an adjusted p-value below 0.05.

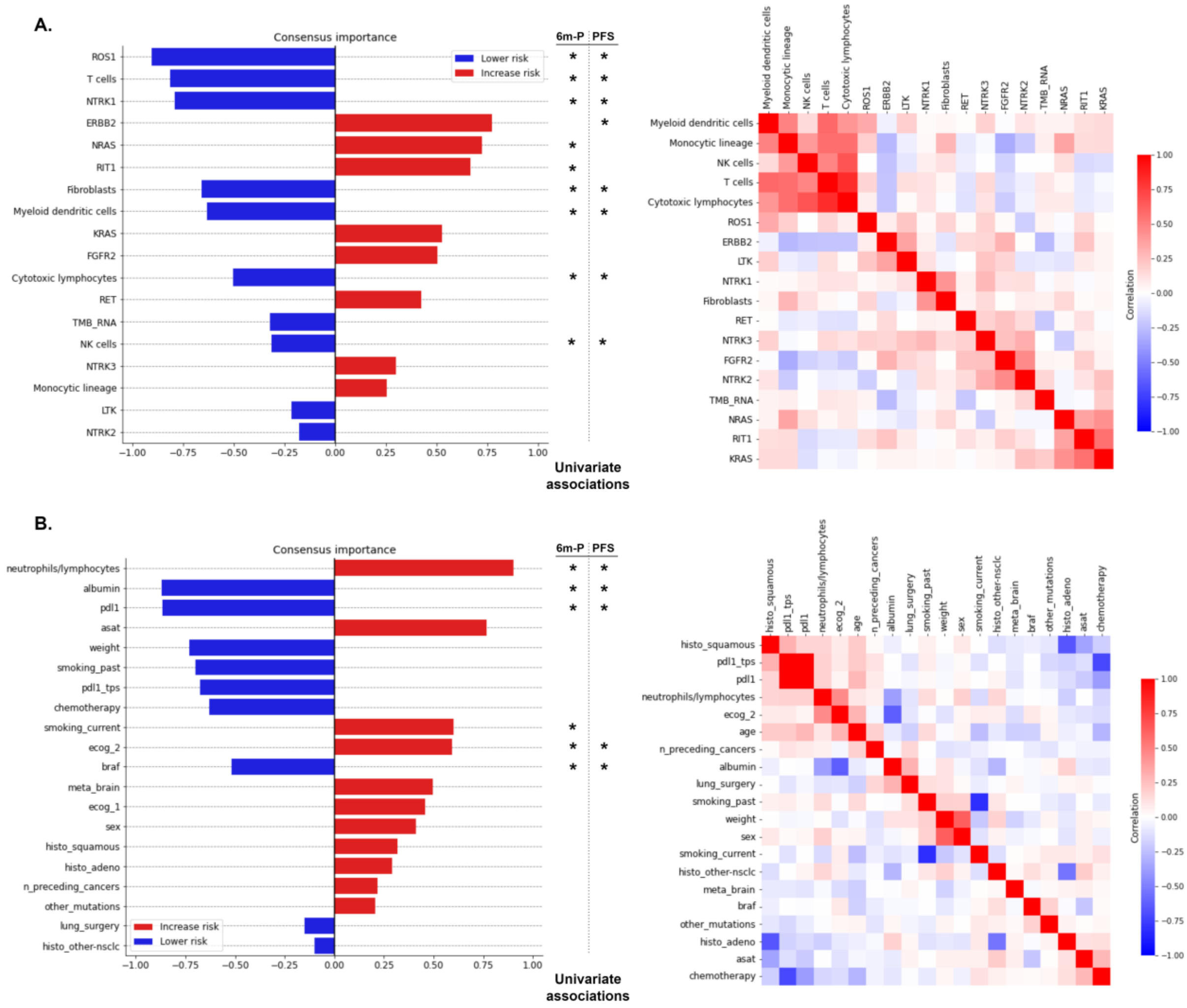

Figure s9: Feature importance ranking for the prediction of progression-free survival, for clinical and transcriptomic modalities.

Feature importance ranking for the prediction of progression-free survival, obtained by aggregating the SHAP values collected from both tasks (PFS and 6-months progression) and both approaches (linear and tree ensemble methods) (see Methods). Features that were significantly associated with 6-months progression (one-sided permutation test with univariate AUCs) after Benjamini-Hochberg (BH) correction ($\alpha = 0.05$) are shown with a * on the left side, while features that were significantly associated with PFS (one-sided permutation test with univariate C-index) after BH correction are annotated with a * on the right side. * corresponds to an adjusted p-value below 0.05. **A.** Consensus feature importance ranking for the RNA data modality (left) and heatmap of Spearman correlations between consensus RNA features (right). **B.** Consensus feature importance ranking for the clinical data modality (left) and heatmap of correlations between consensus clinical features (right). Correlations were evaluated by Spearman correlation coefficients (for continuous feature vs continuous feature), AUCs rescaled to $[-1, 1]$ (for continuous feature vs binary categorical feature), or Matthews correlation coefficient (for binary categorical feature vs binary categorical feature).

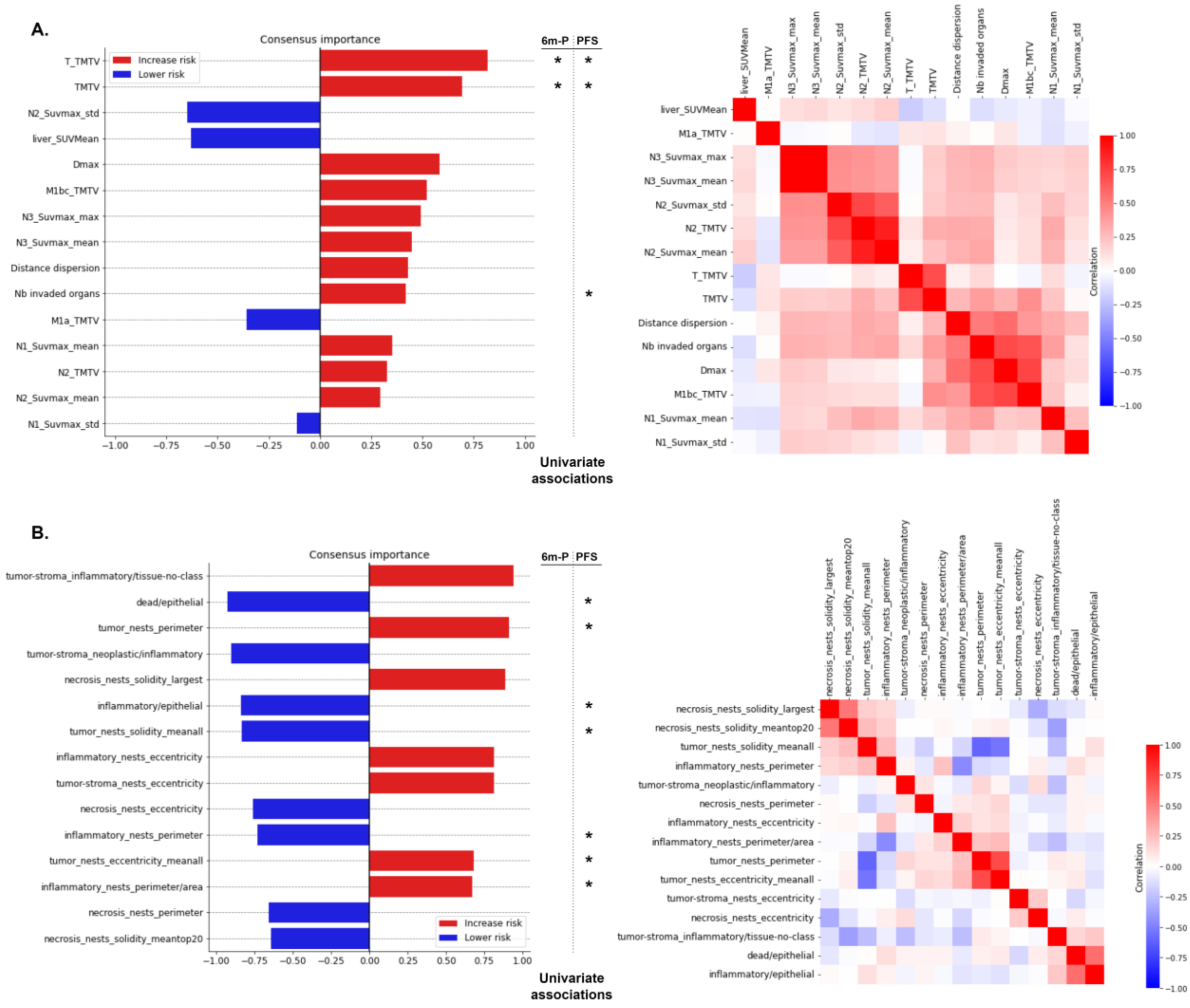

Figure s10: Feature importance ranking for the prediction of progression-free survival, for radiomic and pathomic modalities.

Feature importance ranking for the prediction of progression-free survival, obtained by aggregating the SHAP values collected from both tasks (PFS and 6-months progression) and both approaches (linear and tree ensemble methods) (see Methods). **A.** Consensus feature importance ranking for the radiomic data modality (left) and heatmap of Spearman correlations between consensus radiomic features (right). **B.** Consensus feature importance ranking for the pathomic data modality (left) and heatmap of Spearman correlations between consensus pathomic features (right).

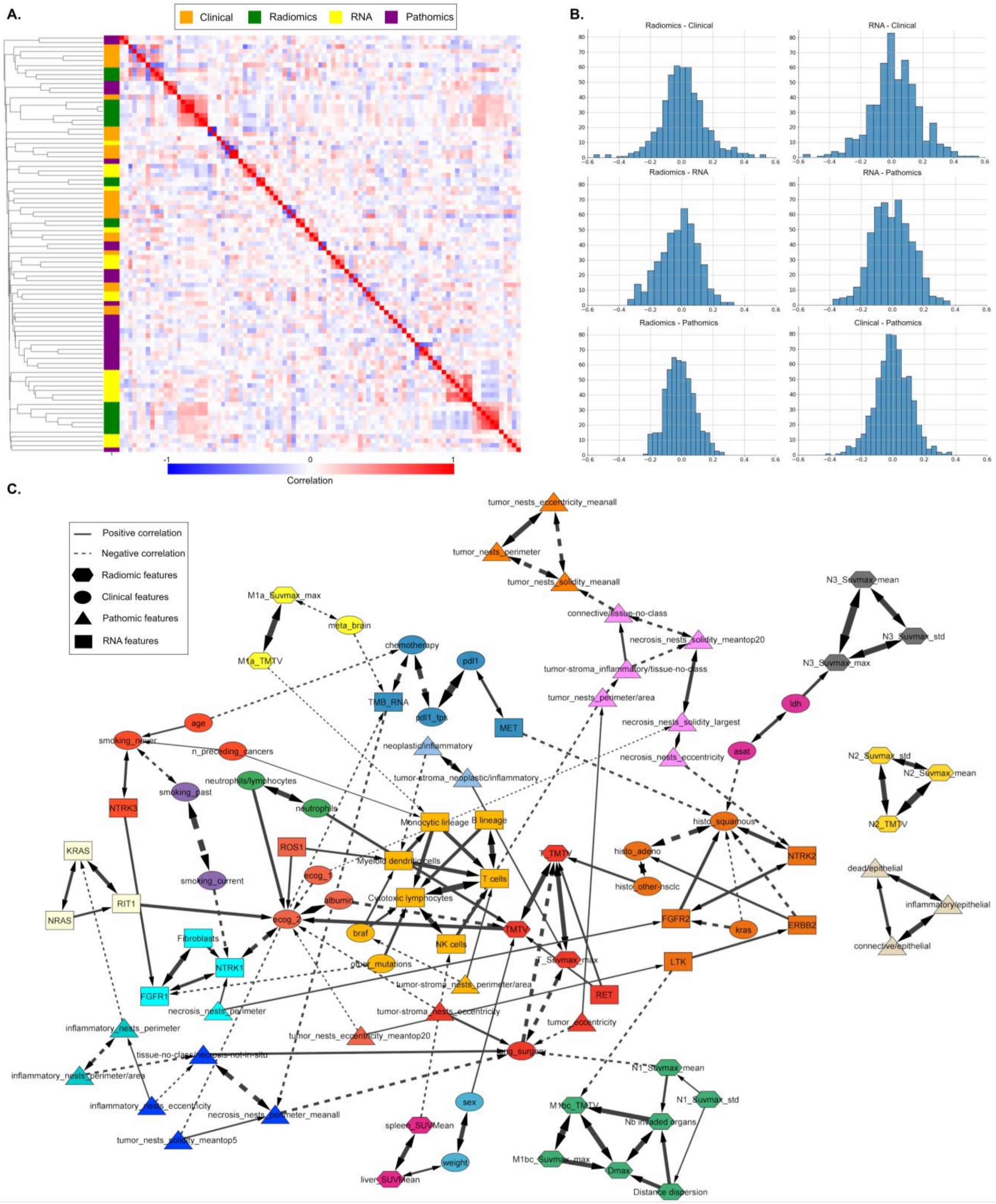

Figure s11: Inter-modal correlations between consensus important features.

A. Heatmap of correlations between consensus important features identified through feature importance analyses (see Methods, Figures 2, s7-s10). Correlations were evaluated across the whole cohort using Spearman correlation coefficients (for continuous vs. continuous features), AUCs rescaled to $[-1,1]$ (for continuous vs. binary categorical features), and Matthews correlation coefficients (for binary categorical vs. binary categorical features). **B.** Distribution of correlation values for pairs of features from different modalities (e.g., RNA vs. clinical features). **C.** Sparse representation of the correlations between

consensus important features using a 2-nearest neighbors graph. The graph was built with a distance metric of $1-|corr|$, where *corr* represents the inter-feature correlation shown in the previous heatmap (see A.). Each node corresponds to a feature, and a directed edge indicates that the target feature is within the 2-nearest neighborhood of the source feature. Node colors represent different graph communities identified using the directed Louvain algorithm.

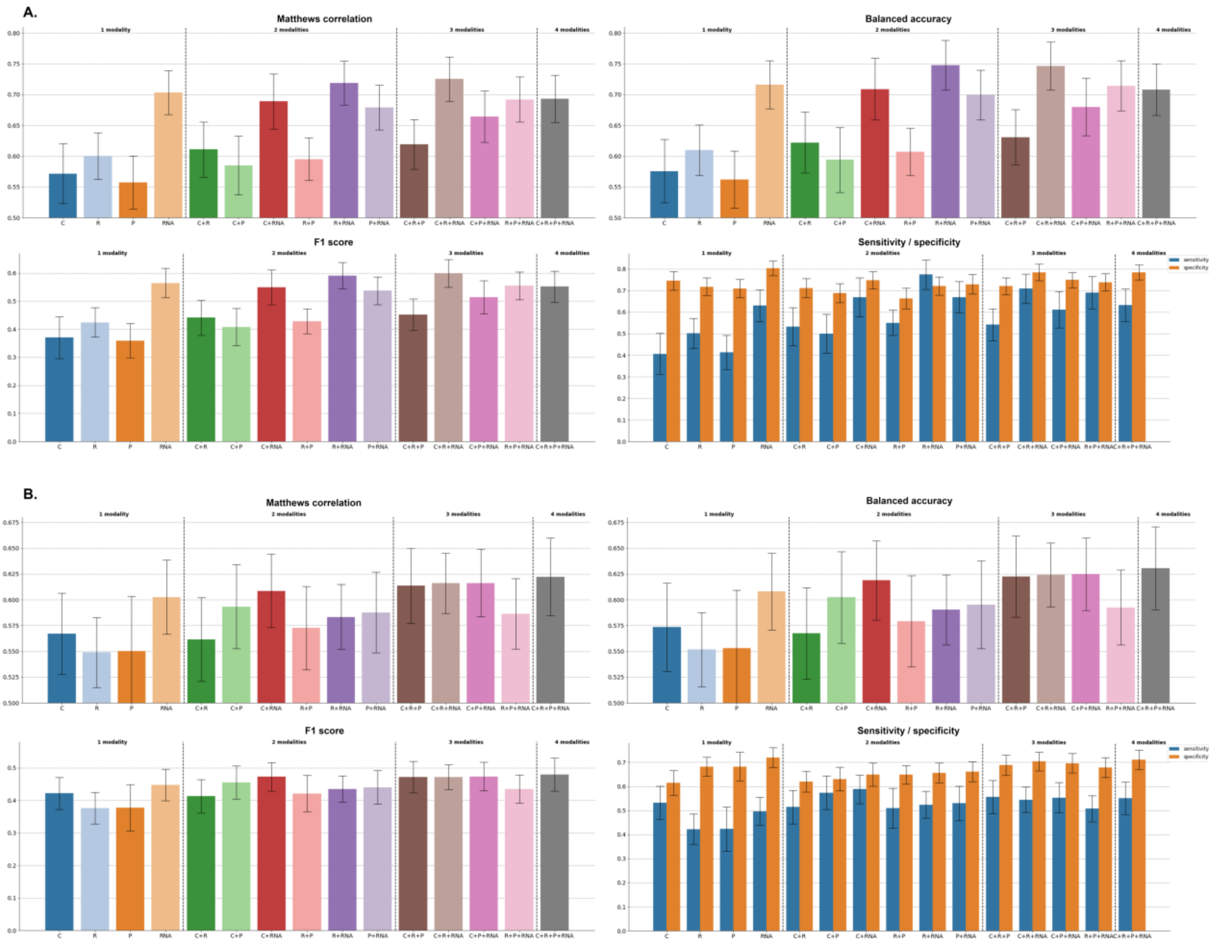

Figure s12: Binary classification performance of all the possible multimodal combinations, with a late fusion strategy and XGBoost.

Binary classification performance of all the possible multimodal combinations, with a late fusion strategy and XGBoost for 1-year death and 6-months progression prediction (C: clinical, R: radiomic, P: pathomic, RNA). The bar height corresponds to the performance metric (balanced Accuracy, Matthews correlation coefficient, F1 score, and sensitivity and specificity) averaged across the 100 cross-validation schemes, and the error bar corresponds to ± 1 standard deviation, estimated across the 100 cross-validation schemes. **A.** Binary metrics associated with the prediction of 1-year death with XGBoost algorithms and estimated with n=77 patients**. B.** Binary metrics associated with the prediction of 6-months progression with XGBoost algorithms and estimated with n=75 patients.

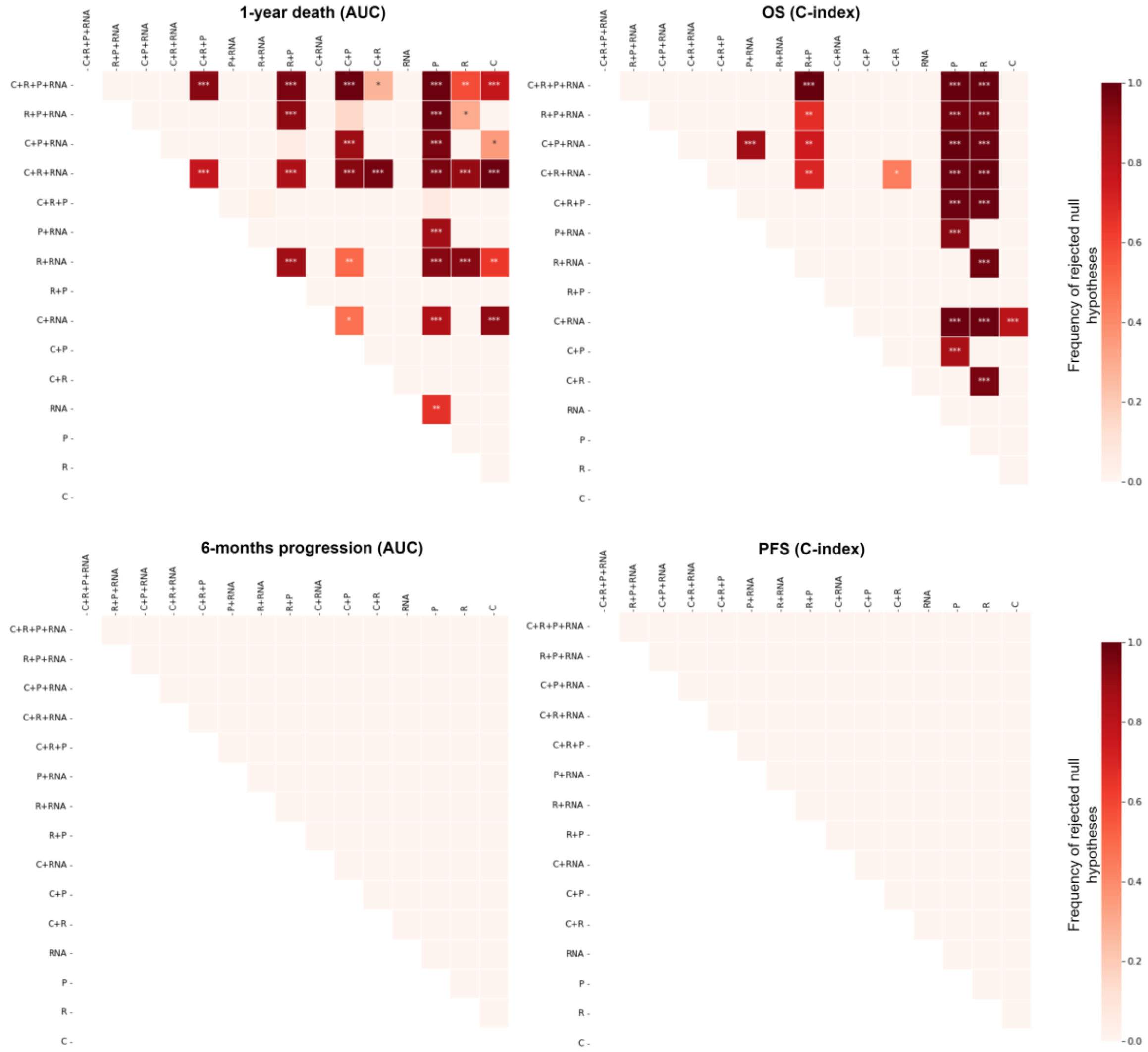

Figure s13: Paired permutation tests to compare multimodal combinations for late fusion strategy with tree ensemble methods.

Paired permutation tests (1) to test for statistically significant differences between the performances (AUC or C-index) of the different multimodal combinations for the late fusion strategy with tree ensemble methods (see Methods). Each element of the heatmap corresponds to the test for the superiority of the row multimodal combination over the column multimodal combination (e.g., the top right corner of the heatmap corresponds to the test of the superiority of the combination with the four modalities over the clinical model). The lower parts of the heatmaps are not shown since they contain no significant comparison. The color scale corresponds to the frequency of statistically significant tests across the 100 cross-validation schemes after Benjamini-Hochberg correction (FDR controlled at level $\alpha = 0.05$). * corresponds to 25 to 50% of significant tests, ** corresponds to 50 to 75% of significant tests, and *** corresponds to more than 75% of significant tests.

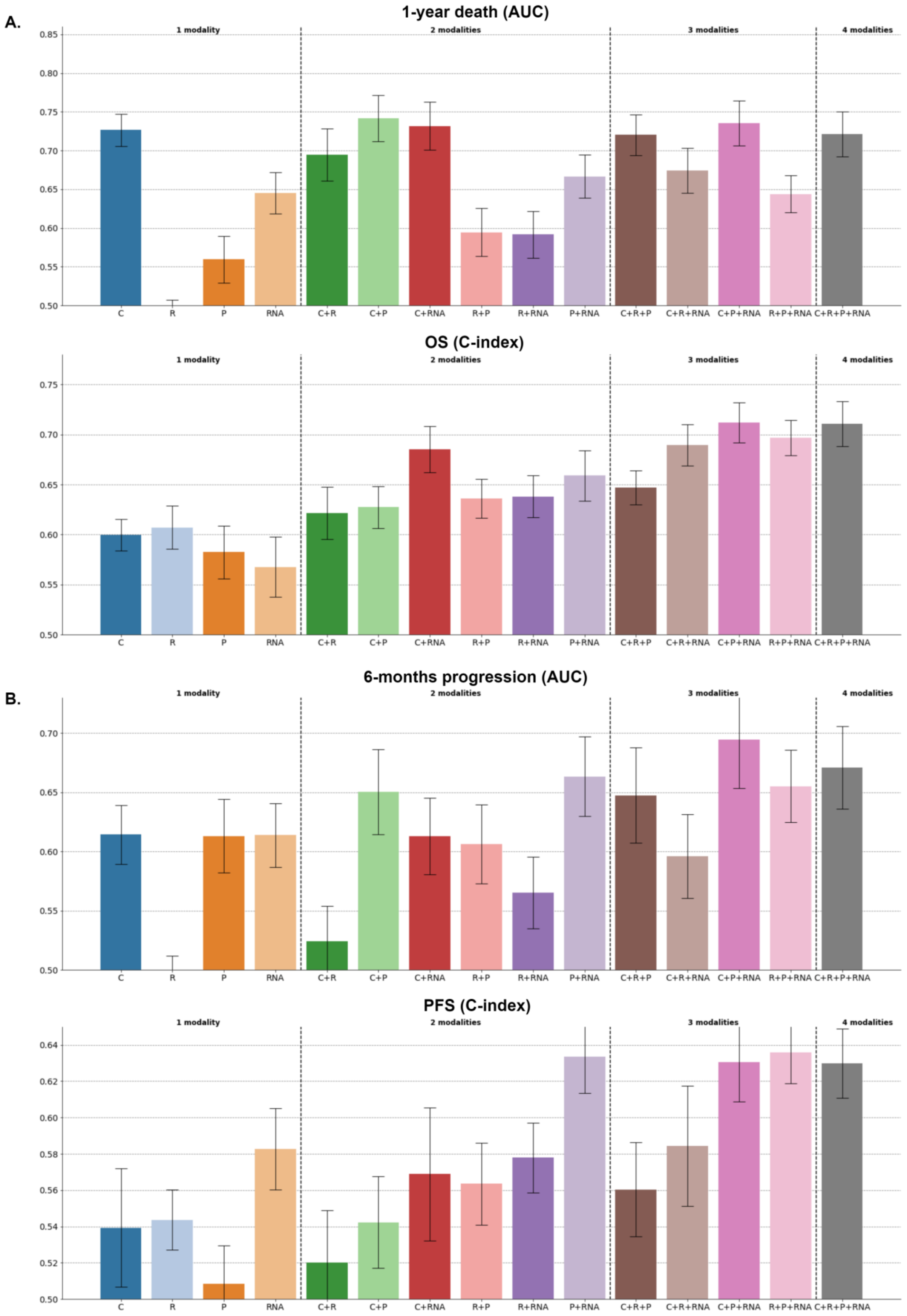

Figure s14: Performance of all the possible multimodal combinations, with a late fusion strategy and linear methods.

A. ROC AUCs associated with the prediction of 1-year death with penalized logistic regression algorithms (top) and estimated with n=77 patients. C-indexes associated with the prediction of OS with penalized Cox's regression algorithms (bottom) and estimated with n=79 patients. **B**. ROC AUCs associated with the prediction of 6-months progression with penalized logistic regression algorithms (top) and estimated with n=75 patients. C-indexes associated with the prediction of PFS with penalized Cox's regression (bottom) and estimated with n=80 patients.

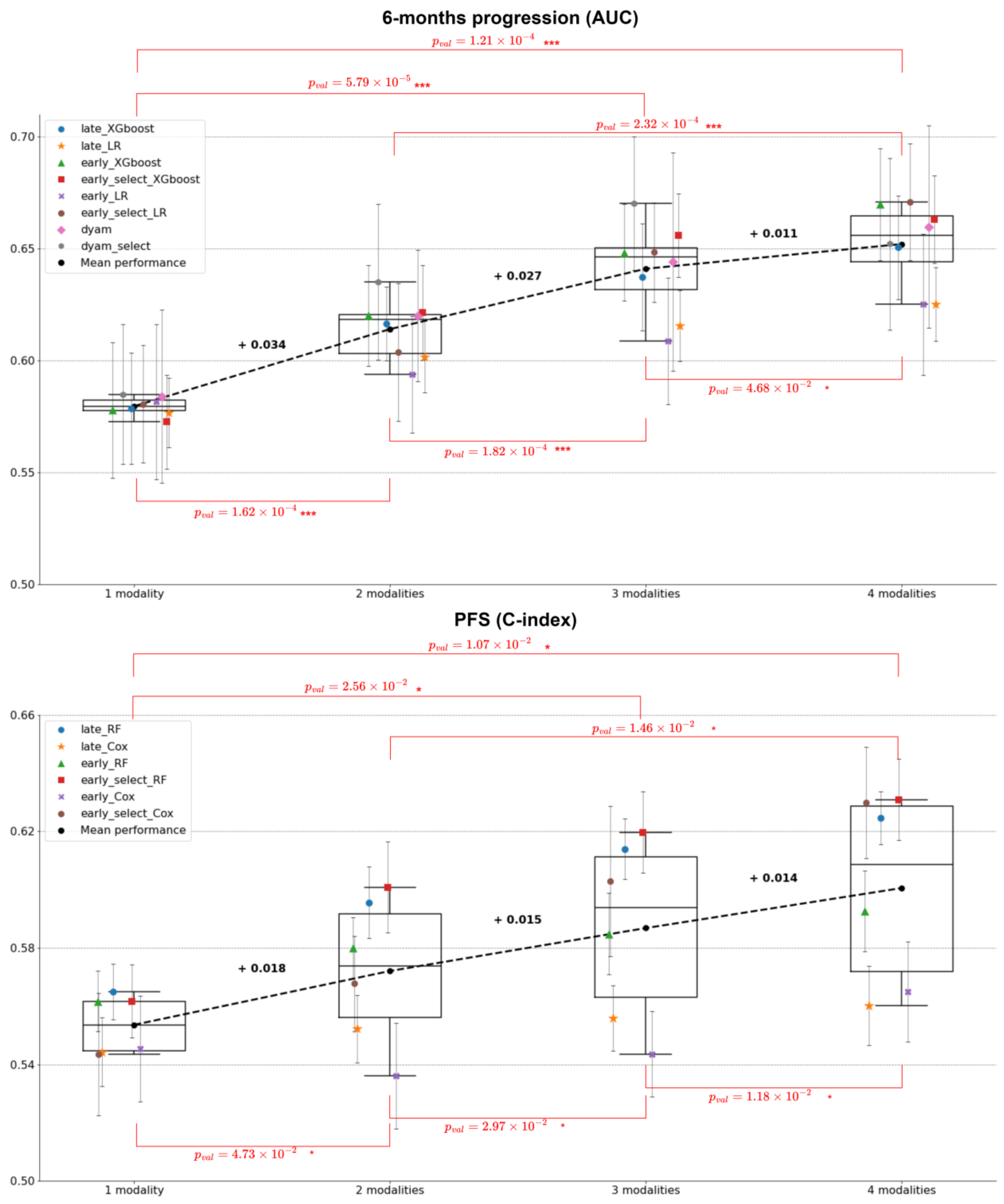

Figure s15: Average performance across all models with 1, 2, 3, and 4 modalities for 6-months progression and PFS.

Markers and error bars correspond to the mean average performance and ± 1 standard deviation respectively, estimated across the 100 cross-validation schemes. The box-and-whisker plots show the three quartiles and the minimum and maximum as whiskers up to 1.5 $\times IQR$ (25%-75%). Mean increases are represented with dashed lines and bold annotations. Red annotations correspond to paired sample t-test p-values to compare the different numbers of integrated modalities (e.g., 1 modality vs 2 modalities), with n_{models}=8 for 6-months progression and n_{models}=6 for PFS. *: 1e-2 <$p_{val}\leq$ 5e-2, **: 1e-4 <$p_{val}\leq$ 1e-3, ***: $p_{val}\leq$1e-4.

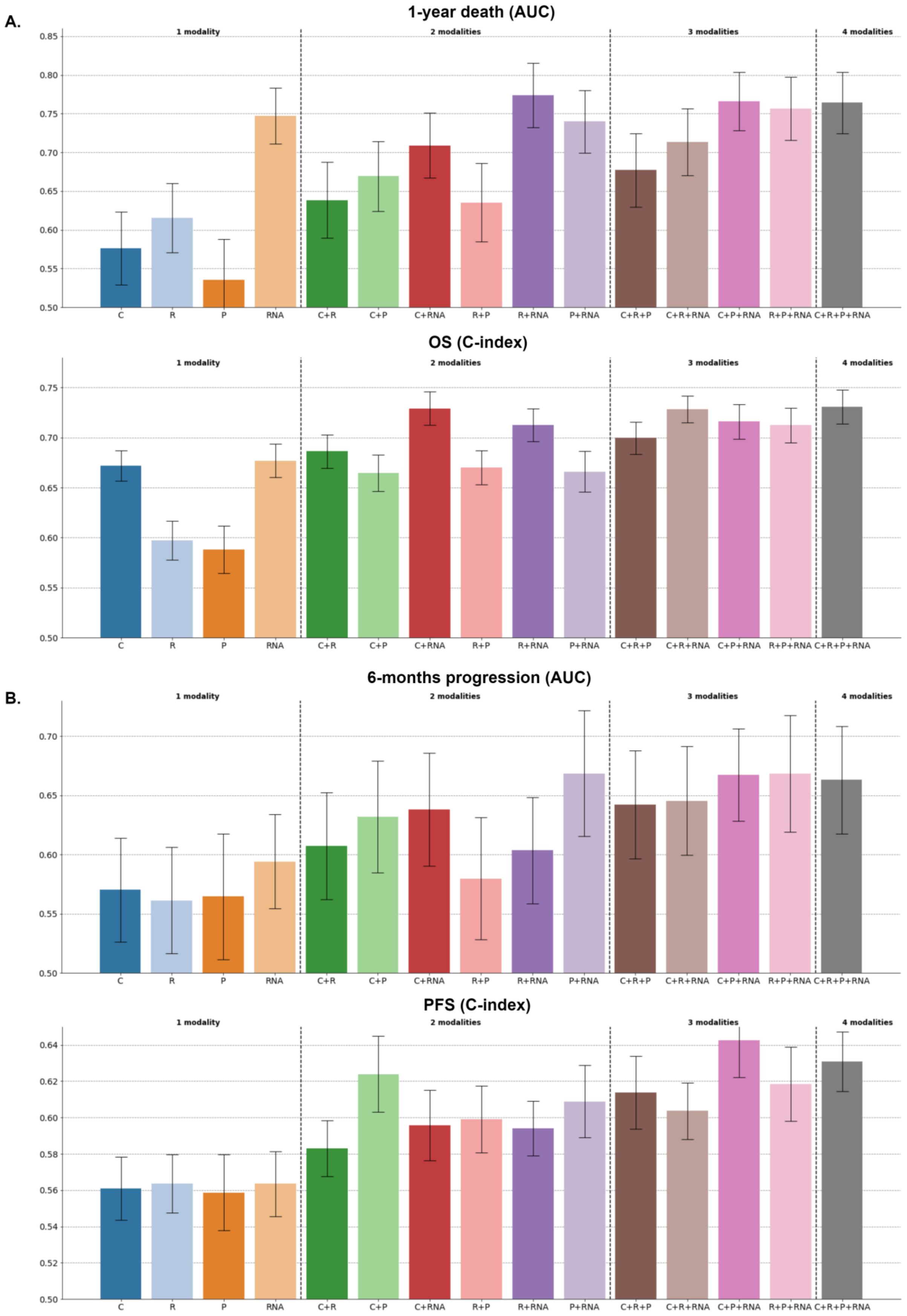

Figure s16: Performance of all the possible multimodal combinations with an early fusion strategy, tree ensemble methods, and a preliminary feature selection step.

A. ROC AUCs associated with the prediction of 1-year death with XGBoost algorithms (top) and estimated with n=77 patients. C-indexes associated with the prediction of OS with Random Forest Survival algorithms (bottom) and estimated with n=79 patients. **B**. ROC AUCs associated with the prediction of 6-months progression with XGBoost algorithms (top) and estimated with n=75 patients. C-indexes associated with the prediction of PFS with Random Survival Forest algorithms (bottom) and estimated with n=80 patients.

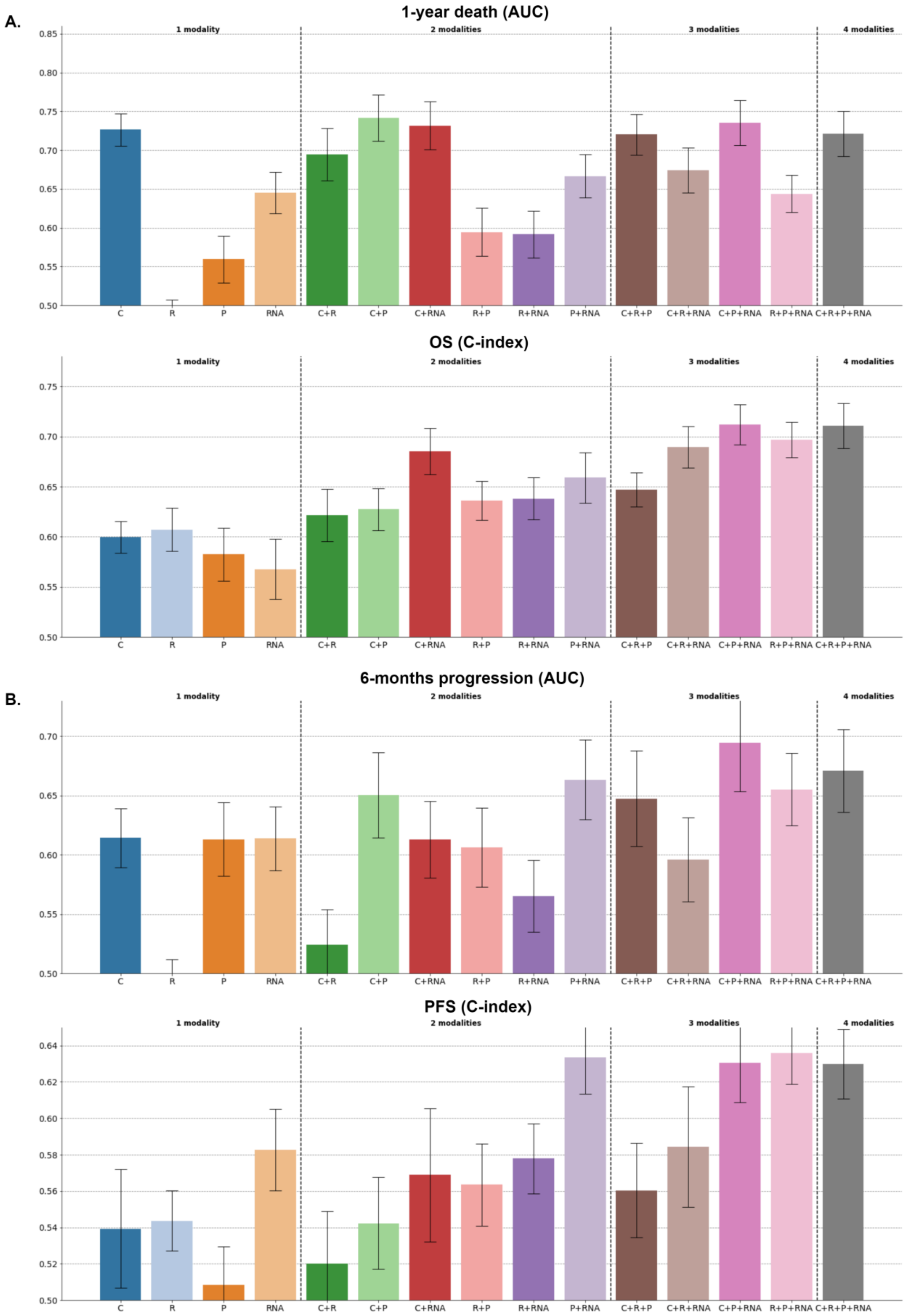

Figure s17: Performance of all the possible multimodal combinations with an early fusion strategy, linear methods, and a preliminary feature selection step.

A. ROC AUCs associated with the prediction of 1-year death with penalized logistic regression algorithms (top) and estimated with n=77 patients. C-indexes associated with the prediction of OS with penalized Cox's regression algorithms (bottom) and estimated with n=79 patients. **B**. ROC AUCs associated with the prediction of 6-months progression with penalized logistic regression algorithms (top) and estimated with n=75 patients. C-indexes associated with the prediction of PFS with penalized Cox's regression (bottom) and estimated with n=80 patients.

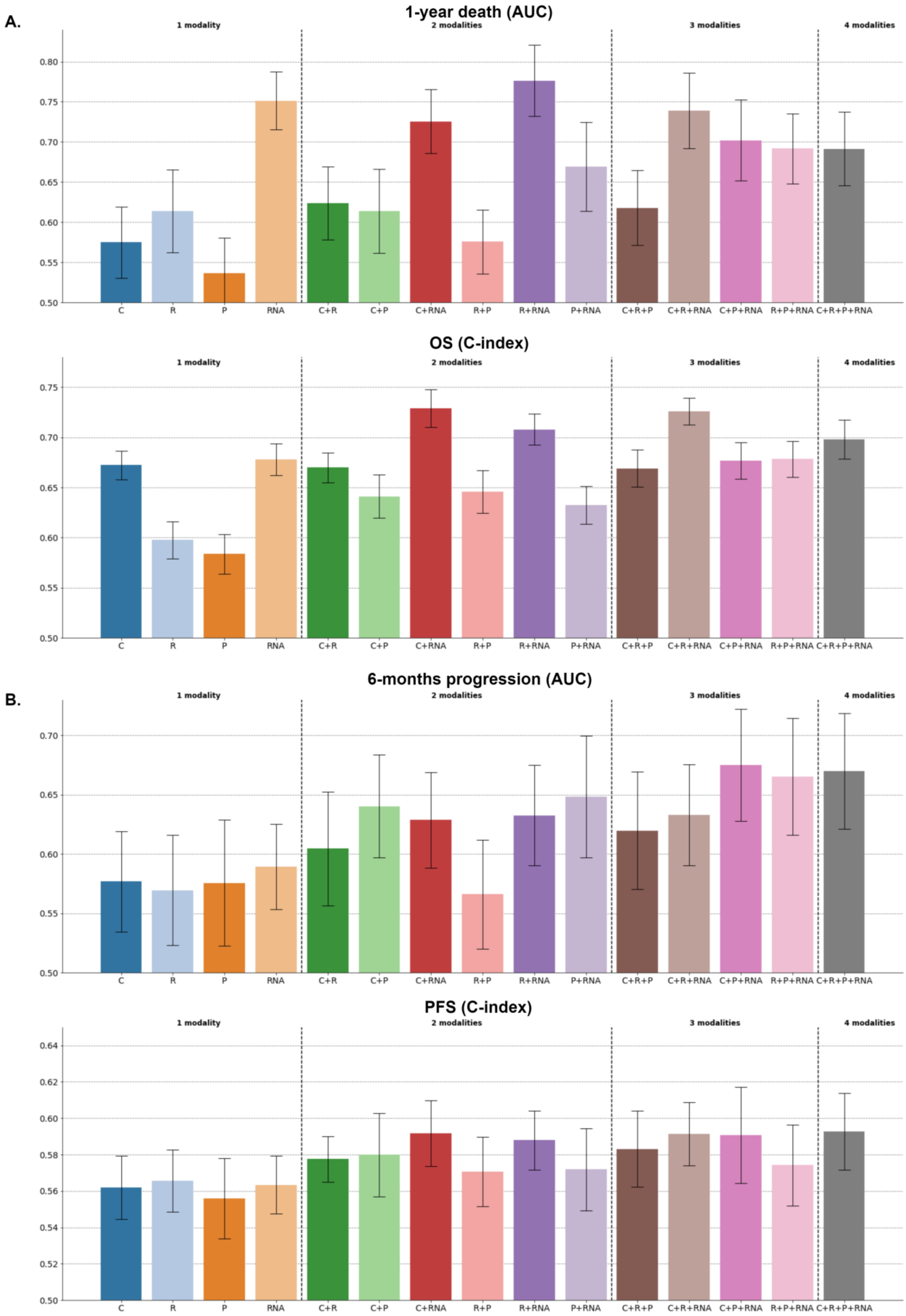

Figure s18: Performance of all the possible multimodal combinations with an early fusion strategy and tree ensemble methods, with no preliminary feature selection step.

A. ROC AUCs associated with the prediction of 1-year death with XGBoost algorithms (top) and estimated with n=77 patients. C-indexes associated with the prediction of OS with Random Forest Survival algorithms (bottom) and estimated with n=79 patients. **B**. ROC AUCs associated with the prediction of 6-months progression with XGBoost algorithms (top) and estimated with n=75 patients. C-indexes associated with the prediction of PFS with Random Survival Forest algorithms (bottom) and estimated with n=80 patients.

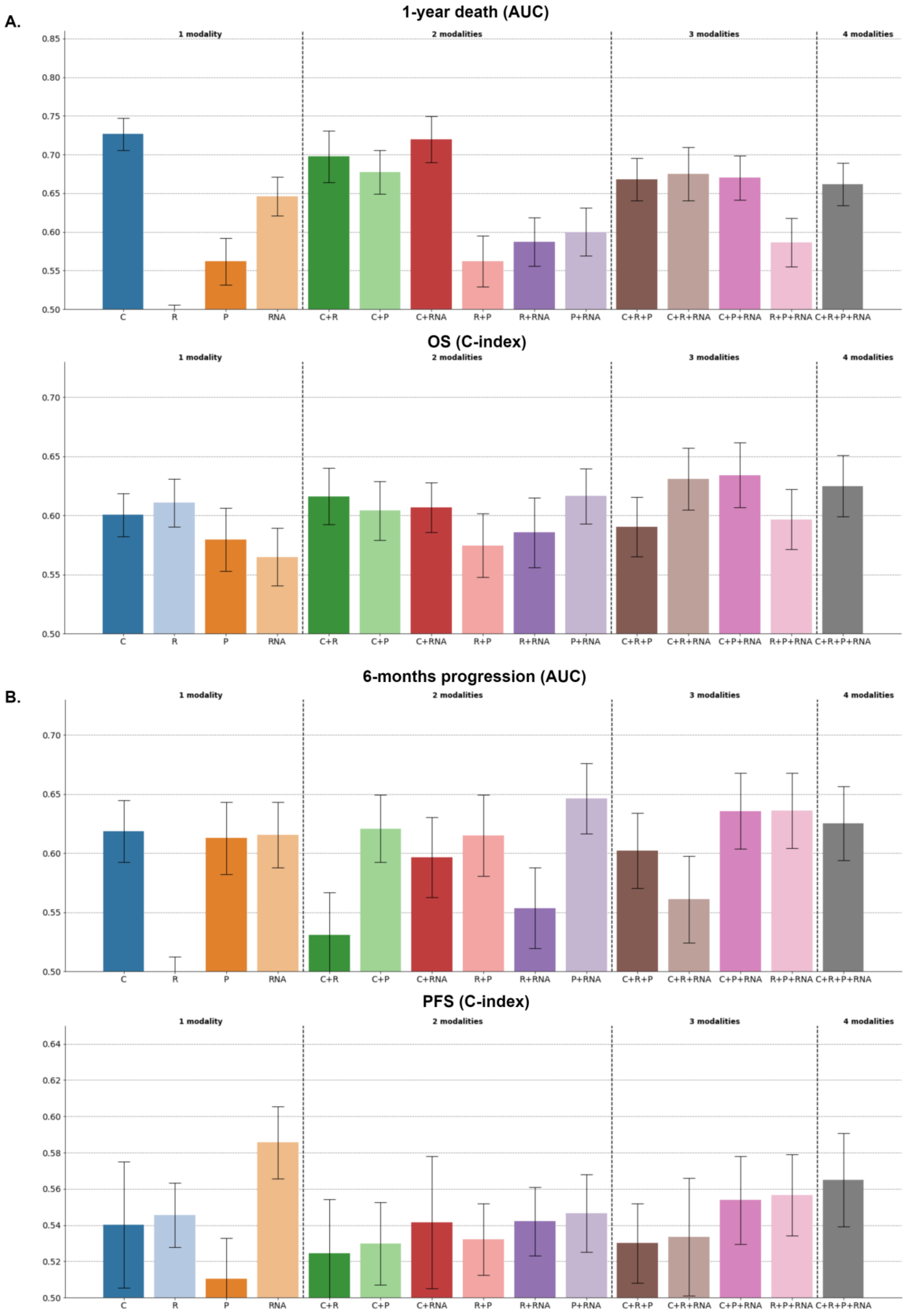

Figure s19: Performance of all the possible multimodal combinations with an early fusion strategy and linear methods, with no preliminary feature selection step.

A. ROC AUCs associated with the prediction of 1-year death with penalized logistic regression algorithms (top) and estimated with n=77 patients. C-indexes associated with the prediction of OS with penalized Cox's regression algorithms (bottom) and estimated with n=79 patients. **B**. ROC AUCs associated with the prediction of 6-months progression with penalized logistic regression algorithms (top) and estimated with n=75 patients. C-indexes associated with the prediction of PFS with penalized Cox's regression (bottom) and estimated with n=80 patients.

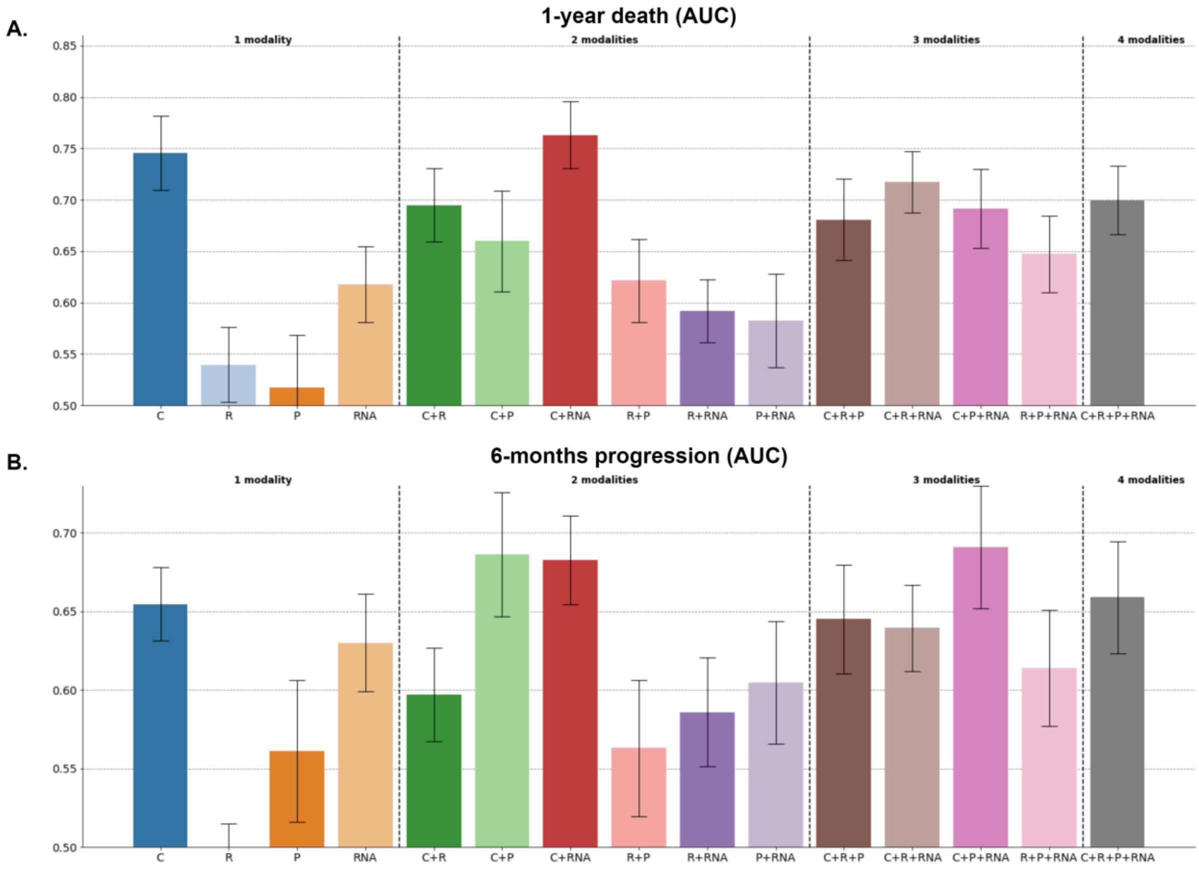

Figure s20: Performance of all the possible multimodal combinations with DyAM strategy with no preliminary feature selection step.

A. ROC AUCs associated with the prediction of 1-year death and estimated with n=77 patients. **B.** ROC AUCs associated with the prediction of 6-months progression and estimated with n=75 patients.

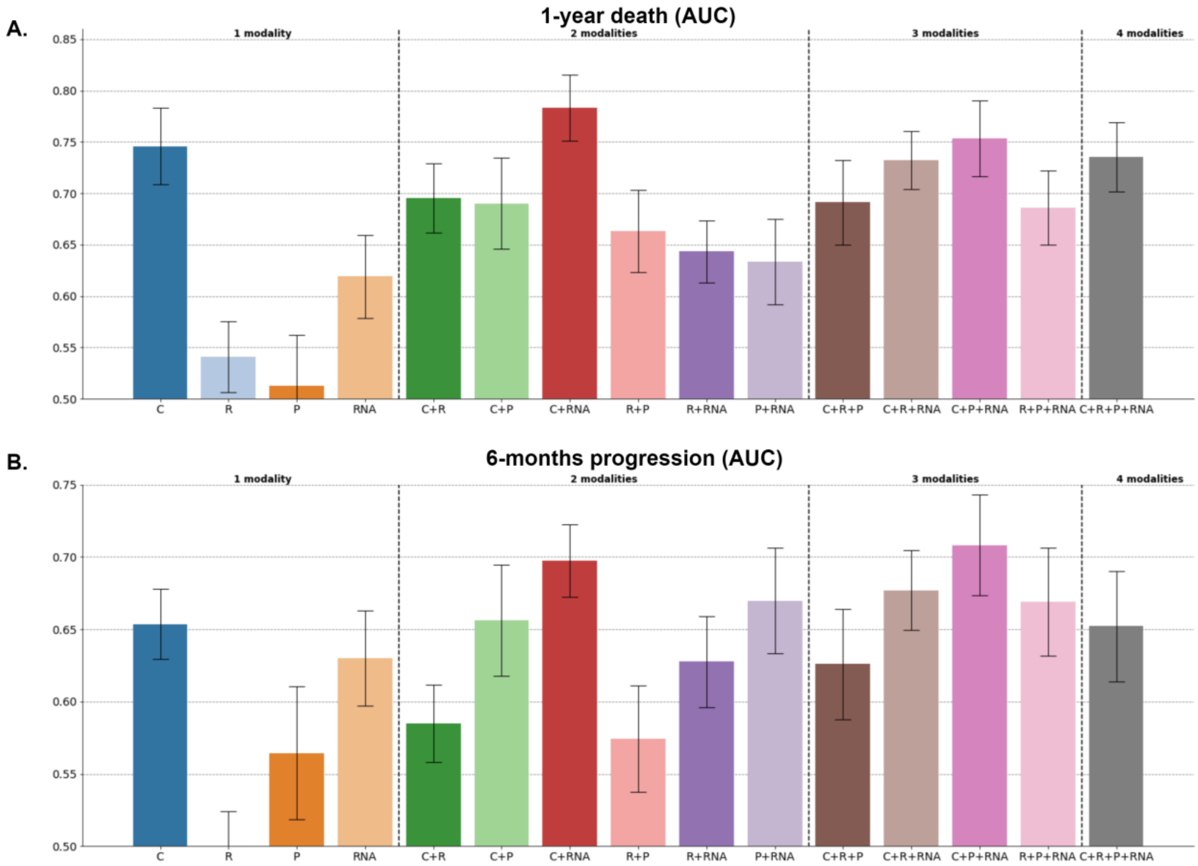

Figure s21: Performance of all the possible multimodal combinations with DyAM strategy and a preliminary feature selection step.

A. ROC AUCs associated with the prediction of 1-year death and estimated with n=77 patients. **B.** ROC AUCs associated with the prediction of 6-months progression and estimated with n=75 patients.

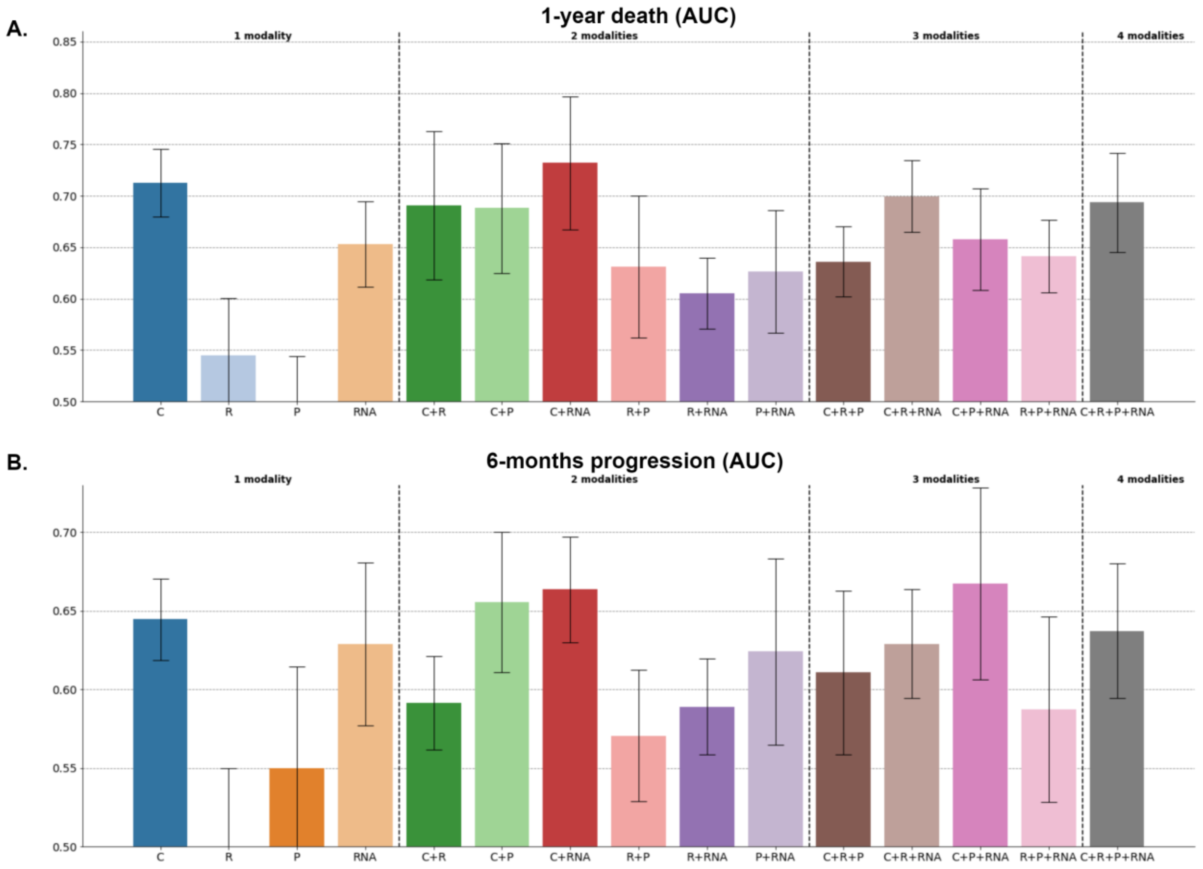

Figure s22: Performance of all the possible multimodal combinations with DyAM strategy and hyperparameter tuning.

The learning rate and the L2 regularization strength were tuned with a grid-search strategy and a nested-cross validation scheme. The experiment was repeated 10 times instead of 100, due to computational constraints. **A.** ROC AUCs associated with the prediction of 1-year death and estimated with n=77 patients. **B.** ROC AUCs associated with the prediction of 6-months progression and estimated with n=75 patients.

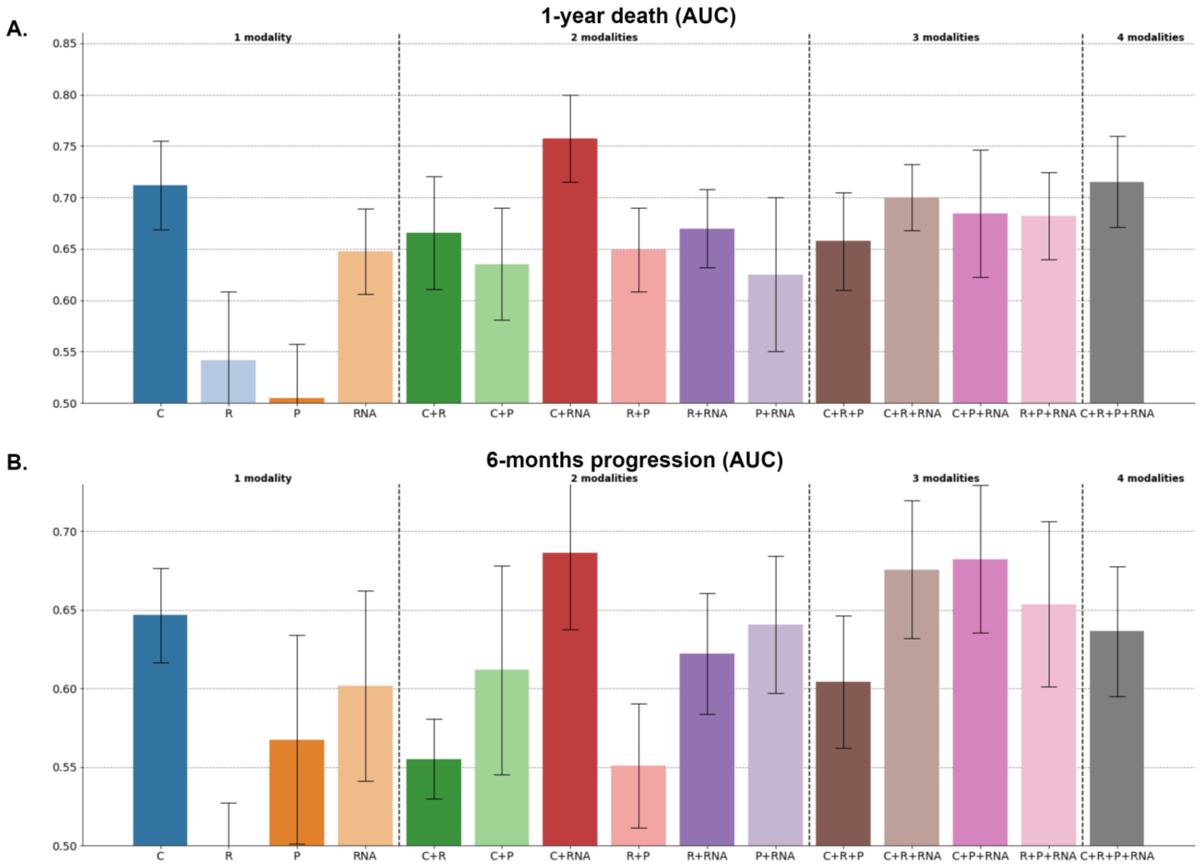

Figure s23: Performance of all the possible multimodal combinations with DyAM strategy, a preliminary feature selection step, and hyperparameter tuning.

The learning rate and the L2 regularization strength were tuned with a grid-search strategy and a nested-cross validation scheme. The experiment was repeated 10 times instead of 100, due to computational constraints. **A.** ROC AUCs associated with the prediction of 1-year death and estimated with n=77 patients. **B.** ROC AUCs associated with the prediction of 6-months progression and estimated with n=75 patients.

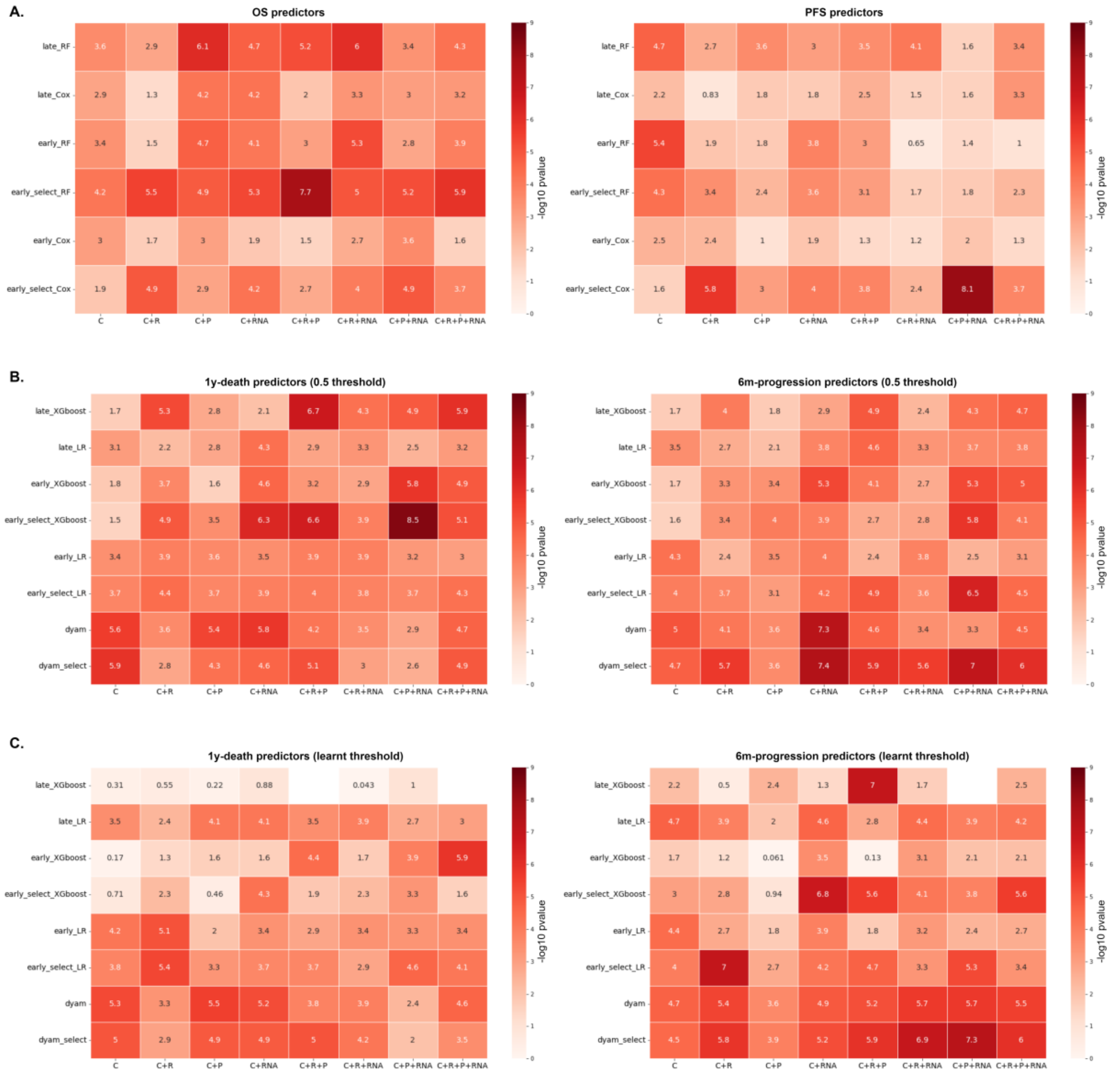

Figure s24: Risk stratification (log-rank p-values) for OS with the predicted multimodal scores.

Unadjusted log-rank p-values ($-\log_{10}(p_{val})$) for the stratification of patients into high-risk and low-risk groups for overall survival, for different predictive tasks (n=265 patients with the 4 targets available for a fair comparison). Only the combinations that include the clinical modality are compared. **A.** Log-rank p-values for models trained to predict OS (left) and models trained to predict PFS (right). Risk-group assignments were determined based on thresholds learned with the model's predictions collected on the training sets of the cross-validation schemes. For PFS models, thresholds were optimized to stratify PFS but were then applied to stratify OS (see Methods). **B.** Log-rank p-values for models trained to predict 1-year death (left) and models trained to predict 6-months progression (right). Risk-group assignments were determined based on a 0.5 threshold. **C.** Log-rank p-values for models trained to predict 1-year death (left) and models trained to predict 6-months progression (right). Risk-group assignments were determined based on thresholds learned with the model's predictions collected on the training sets of the cross-validation schemes. For PFS models, thresholds were optimized to stratify PFS but were then applied to stratify OS.

Supplementary Tables

Table s1: One-sided permutation p-values for the unimodal performance of each data modality.

P-values below 0.05 are indicated in red.

Target (number of patients)		OS (n=79)	1-year death (n=77)	PFS (n=80)	6-months progression (n=75)
Metric		C-index	AUC	C-index	AUC
Clinical	Tree ensembles	< 0.01*	0.07	0.07	0.06
	Linear	0.03	< 0.01*	0.12	0.04
Radiomics	Tree ensembles	0.02	0.07	0.07	0.09
	Linear	0.03	0.58	0.13	0.57
Pathomics	Tree ensembles	0.07	0.17	0.08	0.05
	Linear	0.09	0.18	0.34	0.04
RNA	Tree ensembles	<0.01*	< 0.01*	0.06	0.05
	Linear	0.07	0.06	0.03	0.09

*: p-values below 0.01 could not be estimated with a high precision since the number of permutations was limited to 100 due to computational constraints.

Table s2: List of the 36 transcriptomic signatures associated with immunotherapy and selected from the literature (2).

Category	Name	Cancer type	Immune Checkpoint	Article
Marker genes	CRMA	Melanoma	CTLA-4	10.1016/j.cell.2018.03.026
	CTLA4	Multiple	PD-L1	10.1038/nature14011
	CX3CL1	Multiple	PD-L1	10.1038/nature14011
	CXCL9	Melanoma	PD-L1	10.1016/j.celrep.2020.107873
	CYT	Multiple	PD-1, CTLA-4	10.1016/j.cell.2014.12.033
	EIGS	Multiple	PD-1	10.1172/jci91190
	ESCS	Urothelial	PD-1	10.1038/s41467-018-05992-x
	FTBRS	Multiple	PD-L1	10.1038/nature25501
	HLADRA	Melanoma	PD-1, PD-L1	10.1038/ncomms10582
	HRH1	Multiple	PD-1, PD-L1, CTLA-4	10.1016/j.ccell.2021.11.002
	IFNgamma	Multiple	PD-1	10.1172/jci91190
	Immunopheno	Multiple	PD-1, CTLA-4	10.1016/j.celrep.2016.12.019
	IMPRES	Melanoma	PD-1, CTLA-4	10.1038/s41591-018-0157-9
	IRG	Cervical	PD-1, PD-L1, CTLA-4	10.1080/2162402x.2019.1659094
	MPS	Melanoma	PD-1, CTLA-4	10.1038/s41591-020-0818-3
	PD1	Multiple	PD-1	10.1158/1078-0432.ccr-13-3271
	PDL1	Multiple	PD-1, PD-L1	10.1038/nature14011
	PDL2	Multiple	PD-1	10.1158/1078-0432.ccr-16-1761
	Renal101	Renal Cell	PD-1, PD-L1	10.1038/s41591-020-1044-8
	TIG	Multiple	PD-1	10.1126/science.aar3593
	TLS	Melanoma	PD-1, CTLA-4	10.1038/s41586-019-1914-8
	TME	Gastric	PD-1, PD-L1, CTLA-4	10.1158/2326-6066.cir-18-0436
GSEA	APM	Clear Cell Renal Cell	PD-1	10.1186/s13059-016-1092-z
	CECMdown	Multiple	PD-1	10.1038/s41467-018-06654-8
	CECMup	Multiple	PD-1	10.1038/s41467-018-06654-8
	IIS	Clear Cell Renal Cell	PD-1	10.1186/s13059-016-1092-z
	IMS	Gastric	PD-1, PD-L1	10.1038/s41525-021-00249-x
	IPRES	Multiple	PD-1	10.1016/j.cell.2016.02.065
	MFP	Multiple	PD-1, PD-L1, CTLA-4	10.1016/j.ccell.2021.04.014
	MIAS	Melanoma	PD-1	10.1038/s41467-021-27651-4
	PASSPRE	Melanoma	PD-1	10.1038/s41467-021-26299-4
	TIS	Clear Cell Renal Cell	PD-1	10.1186/s13059-016-1092-z
Deconvolution	CD8T_ CIBERSORT	Multiple	PD-1	10.1038/nature13954
	CD8T_ MCPcounter	Multiple	PD-1	10.1038/nature13954
	CD8T_ Xcell	Multiple	PD-1	10.1038/nature13954
	Immuno_ CIBERSORT	Melanoma	PD-1	10.18632/aging.102556

Supplementary References

1. Bandos AI, Rockette HE, Gur D. A permutation test sensitive to differences in areas for comparing ROC curves from a paired design. Stat Med. 2005 Sep 30;24(18):2873-93.
2. Kang H, Zhu X, Cui Y, Xiong Z, Zong W, Bao Y, Jia P. A Comprehensive Benchmark of Transcriptomic Biomarkers for Immune Checkpoint Blockades. Cancers. 2023; 15(16):4094.
